# PRMT5 promotes DNA repair through methylation of 53BP1 and is regulated by Src-mediated phosphorylation

**DOI:** 10.1038/s42003-020-01157-z

**Published:** 2020-08-05

**Authors:** Jee Won Hwang, Su-Nam Kim, Nayeon Myung, Doona Song, Gyoonhee Han, Gyu-Un Bae, Mark T. Bedford, Yong Kee Kim

**Affiliations:** 1 Research Institute of Pharmaceutical Sciences, College of Pharmacy, Sookmyung Women’s University, Seoul, 04310 Republic of Korea; 2 Natural Product Research Institute, Korea Institute of Science and Technology, Gangneung, 25451 Republic of Korea; 3 Department of Biotechnology, Department of Biomedical Sciences, Yonsei University, Seoul, 03722 Republic of Korea; 4 Department of Epigenetics and Molecular Carcinogenesis, The University of Texas MD Anderson Cancer Center, Smithville, TX 78957 USA

**Keywords:** Methylation, Phosphorylation, DNA damage and repair

## Abstract

PRMT5 participates in various cellular processes, including transcription regulation, signal transduction, mRNA splicing, and DNA repair; however, its mechanism of regulation is poorly understood. Here, we demonstrate that PRMT5 is phosphorylated at residue Y324 by Src kinase, a negative regulator of its activity. Either phosphorylation or substitution of the Y324 residue suppresses PRMT5 activity by preventing its binding with the methyl donor S-adenosyl-L-methionine. Additionally, we show that PRMT5 activity is associated with non-homologous end joining (NHEJ) repair by methylating and stabilizing p53-binding protein 1 (53BP1), which promotes cellular survival after DNA damage. Src-mediated phosphorylation of PRMT5 and the subsequent inhibition of its activity during the DNA damage process blocks NHEJ repair, leading to apoptotic cell death. Altogether, our findings suggest that PRMT5 regulates DNA repair through Src-mediated Y324 phosphorylation in response to DNA damage.

## Abstract (web summary)

Hwang et al. show that the activity of PRMT5 methyltransferase is regulated by Src kinase-mediated phosphorylation at Y324 in response to DNA damage. They also show that PRMT5 participates in NHEJ repair by regulating 53BP1 protein levels and is critical for cellular survival after DNA damage.

## Introduction

Arginine methylation is an abundant post-translational modification (PTM), which is catalyzed by protein arginine methyltransferases (PRMTs). To date, nine PRMTs have been characterized that produce three types of methylarginines: monomethylarginine, asymmetric dimethylarginine (ADMA), and symmetric dimethylarginine (SDMA). PRMT5 is a type II methyltransferase that catalyzes mono- or symmetric dimethylation of arginine residues on its substrate proteins^1–3^. Many studies have focused on the epigenetic role of PRMT5 that is executed through the symmetric dimethylation of histone proteins at H3R8, H4R3, and H2AR3 sites, resulting in suppression or activation of gene expression^4–6^. In addition, several studies have shown that PRMT5 also targets various non-nucleosomal proteins, including components of spliceosome, PIWI proteins, Rad9, REL-A, EGFR, CRAF, E2F-1, and p53^7–15^, thereby implicating it in multiple cellular signaling events. Furthermore, it has been reported that upregulation of PRMT5 is closely associated with tumor progression and poor prognosis in several human cancers, including breast, gastric, colorectal, ovarian, leukemia, and lymphoma^3,16^. Although the cellular functions of PRMT5 have been extensively studied, little is known about how its methyltransferase activity is regulated. A single report shows that PRMT5 is phosphorylated and inhibited by the constitutively active Janus Kinase 2 (JAK2) mutant JAK2V617F, but not by wild type JAK2^17^. In this case, JAK2V617F-mediated phosphorylation of PRMT5 disrupts its association with methylosome protein 50 (MEP50)/WDR77, a well-known co-activator required for PRMT5 activity. JAK2V617F-dependent regulation of PRMT5 has provided an understanding of the molecular pathogenesis of myeloproliferative neoplasm; however, the regulatory mechanism for PRMT5 activity in normal physiological processes is yet to be unraveled.

DNA double-strand break (DSB) repair processes are critical for the maintenance of genomic stability, and they protect cells against exogenous and endogenous DNA damage stresses^18,19^. Defects to DNA repair mechanism lead to mutagenic diseases, including cancer and neurological dysfunction^20^. DSB repair is accomplished through two main pathways: homologous recombination (HR) and non-homologous end joining (NHEJ). HR is error-free, but requires a sister chromatid as a template and, therefore, only occurs during late S/G2, while NHEJ is error-prone and can occur throughout the cell cycle^21,22^. There is evidence that an increase in error-prone NHEJ is linked to chromosomal instability in human cancers, which is considered to be an attractive therapeutic target^23–25^. The NHEJ-mediated repair pathway is primarily controlled by a protein complex containing p53 binding protein 1 (53BP1), Rif1, PTIP, and Rev7^21^. After DSBs, 53BP1 is rapidly recruited into damaged DNA lesions, where it forms nuclear foci and transduces DNA repair processes. The disruption of signaling pathways that regulate the targeting of 53BP1 may be a good way to sensitize cancer cells to radiotherapy.

In this study, we show that PRMT5 activity is regulated by Src kinase-mediated phosphorylation at Y324, a critical residue for its enzymatic activity. This phosphorylation occurs in response to DNA damage stresses. PRMT5 participates in the NHEJ repair process by regulating 53BP1 protein levels, and consequently, it is critical for cellular survival after DNA damage. Thus, our findings demonstrate the importance of PRMT5 activity in the DNA repair process, which is regulated by Src kinase-mediated Y324 phosphorylation.

## Results

### PRMT5 is phosphorylated at Y324 by Src kinase

**Fig. 1:**
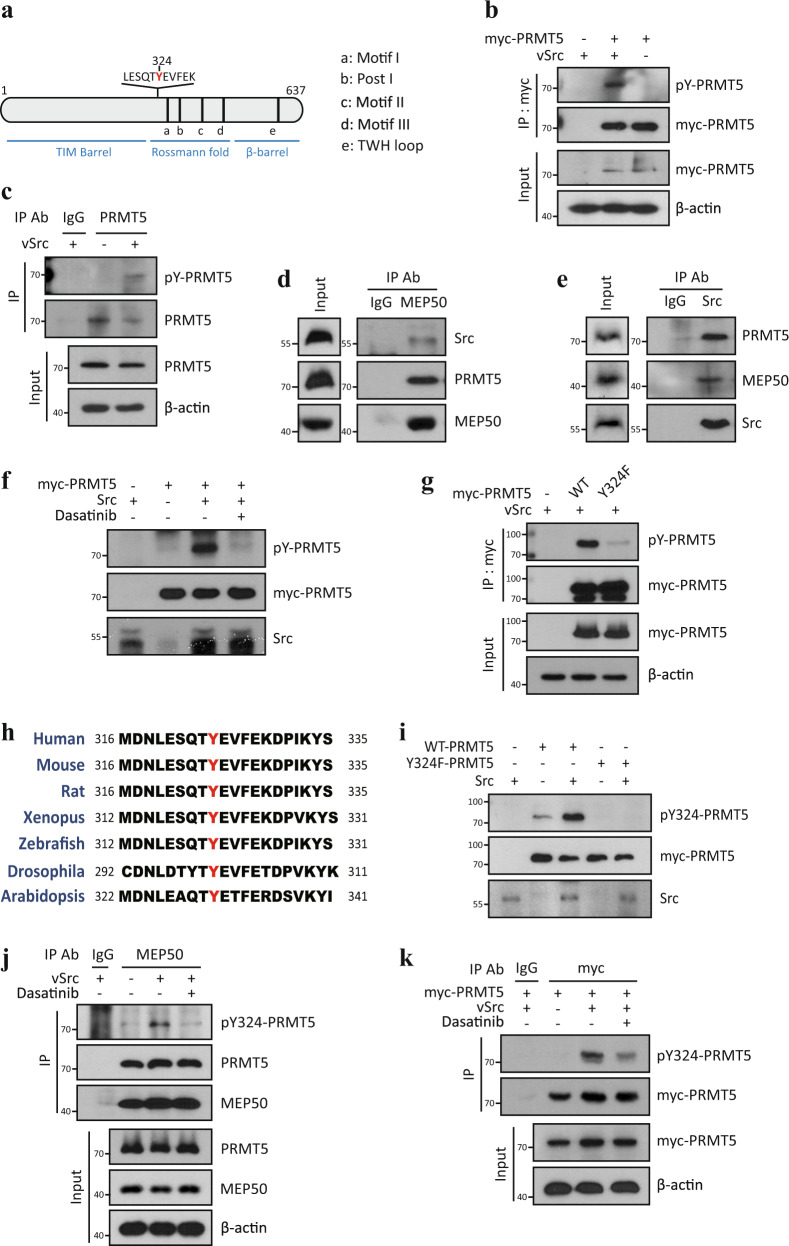
PRMT5 is phosphorylated at Y324 residue by Src kinase. **a** A schematic representation of the domain structure and the predicted phosphorylated residue (Y324) of human PRMT5. The vertical black lines indicate five conserved PRMT signature motifs (a, Motif I: VLD/EVGXGXG; b, Post I: V/IXG/AXD/E; c, Motif II: F/I/VDI/L/K; d, Motif III: LR/KXXG; e, THW loop). **b** IP-Western analysis of myc-PRMT5 tyrosine phosphorylation. HEK293T cells were transfected with myc-PRMT5 alone or in combination with vSrc. Whole-cell lysates were subjected to immunoprecipitation using a c-myc antibody, followed by immunoblotting with phospho-Tyr specific antibody (pY100). **c** Tyrosine phosphorylation of endogenous PRMT5. HEK293T cells were transfected with vSrc plasmid and then endogenous PRMT5 was immunoprecipitated using PRMT5 antibody. Phosphorylated PRMT5 was detected using pY100 antibody. **d**, **e** Physical interaction between endogenous PRMT5 and Src kinase. Western blot assay of PRMT5 and Src kinase using the anti-MEP50 (**d**) or anti-Src kinase (**e**) immunoprecipitants from U2OS cells. **f** In vitro Src kinase assay. Myc-PRMT5 purified from HEK293T cells, Src kinase from vSrc-transfected A549 cells, and ATP were all incubated with or without 1 μM dasatinib. Tyrosine phosphorylation of myc-PRMT5 was detected by immunoblotting using pY100 antibody. **g** Y324 phosphorylation of myc-PRMT5. U2OS cells were co-transfected with myc-PRMT5 (WT or Y324F) and vSrc plasmids, and then immunoprecipitated using c-myc antibody. The immunoprecipitants and input lysates were immunoblotted as described. Tyrosine phosphorylation of myc-PRMT5 was detected by pY100 antibody. **h** Amino acid alignment of PRMT5 from different species. Tyrosine 324 residue (of human PRMT5) is highlighted in red. **i** In vitro Src kinase assay. WT or Y324F myc-PRMT5 was incubated with Src kinase. The phosphorylation of myc-PRMT5 was determined by immunoblotting using pY324-PRMT5 antibody. **j**, **k** Src kinase-dependent Y324 phosphorylation of PRMT5. U2OS cells were transfected with vSrc alone (**j**) or myc-PRMT5 and vSrc (**k**) for 1 day and then incubated with 1 μM dasatinib for 4 h. Endogenous PRMT5 (**j**) or myc-PRMT5 (**k**) was immunoprecipitated using MEP50 or c-myc antibody, respectively. The immunoblots in (**b**–**g**, **i**–**k**) are representative of three independent experiments with similar results, respectively.

To elucidate the regulatory mechanism of PRMT5 activity, we first investigated whether there are motifs that could be phosphorylated on PRMT5. Using the web-based database Scansite (http://scansite3.mit.edu), which predicts protein motifs that are likely to be phosphorylated by specific protein kinases, we found that several kinases, including Src kinase, PDGFR, AKT, and ATM, are predicted as the responsible enzymes for phosphorylation of PRMT5 at Y324, T634, and S446 residues, respectively (Supplementary Fig. 1a). Furthermore, an analysis of the curated phosphosite database reveals that Y324 phosphorylation has been reported by over 70 independent mass spectrometry studies, making it one of the most common reported post-translational modification on a PRMT family member. In this study, we focus on the Y324 residue because it is closely located to Motif I (Fig. 1a), which is one of the five AdoMet binding motifs common to the PRMTs family^26^. Tyrosine phosphorylation of PRMT5 was clearly detected when viral Src kinase (vSrc, constitutively active form due to C-term truncation of cSrc (Δ527–533)) was overexpressed in HEK293T cells (Fig. 1b and c). To confirm whether PRMT5 is a novel substrate of Src kinase, we examined the physical interaction between PRMT5 and Src kinase using co-IP assay. Endogenous Src kinase binds to ectopically overexpressed myc-PRMT5 protein (Supplementary Fig. 1b). To strengthen this, we attempted to investigate the interaction of the endogenous proteins using anti-PRMT5 antibody. However, owing to the very low immunoprecipitation efficiency of PRMT5 antibodies, we failed to precipitate sufficient endogenous PRMT5. Instead, we performed co-IP assay using a MEP50 antibody, which is a well-known co-activator of PRMT5^3,26^. This successfully precipitated a large amount of endogenous PRMT5 protein and simultaneously Src kinase protein (Fig. 1d). This interaction between Src kinase and PRMT5 was also validated by a reciprocal co-IP assay using the Src kinase antibody (Fig. 1e), thus demonstrating that endogenous Src kinase binds to PRMT5-MEP50 complex in vivo.

To establish whether PRMT5 is directly phosphorylated by Src kinase, we performed an in vitro kinase assay. As shown in Fig. 1f, Src kinase was able to strongly phosphorylate PRMT5 in vitro; however, treatment with dasatinib, a Src family kinase inhibitor completely inhibited this phosphorylation. In addition, the tyrosine phosphorylation of PRMT5 by vSrc was completely abolished in the Y324F mutant of PRMT5 (Fig. 1g), indicating that the Y324 residue of PRMT5 is the major phosphorylation site for Src kinase. Protein sequence alignment analysis reveals that the Y324 residue of PRMT5 is highly conserved in evolution (Fig. 1h); however, it is not found in other PRMT family members (Supplementary Fig. 1c), implying that the Y324 phosphorylation may selectively control the cellular functions of PRMT5. In order to study the biological role of the Y324 phosphorylation of PRMT5, we developed a phospho-Y324 PRMT5 specific antibody, pY324-PRMT5 (Supplementary Fig. 1d). Compared to the pY100 antibody, our pY324-PRMT5 antibody recognized the PRMT5 phosphorylation by Src kinase more effectively (Supplementary Fig. 1e). The Y324 phosphorylation was rarely detected in the Y324F mutant, whereas it occurred in the GRAA mutant (an enzyme dead form of PRMT5 where the G367-R368 residues are substituted with alanine, respectively^27^) with the intact Y324 residue (Supplementary Fig. 1f). Also, the immunopositive western band by pY324-PRMT5 antibody was removed by CIP (calf intestinal alkaline phosphatase), validating the efficiency of this antibody (Supplementary Fig. 1g). The pY324-PRMT5 antibody clearly detected Src kinase-mediated Y324 phosphorylation in wild type PRMT5 (WT), but not in Y324F mutant (Fig. 1i) in vitro. In addition, we also confirmed that the Y324 phosphorylation occurs in a Src kinase-dependent manner in vivo (Fig. 1j and k). All these results corroborate that PRMT5 gets phosphorylated at Y324 by Src kinase.

### The Y324 phosphorylation of PRMT5 suppresses its activity

**Fig. 2:**
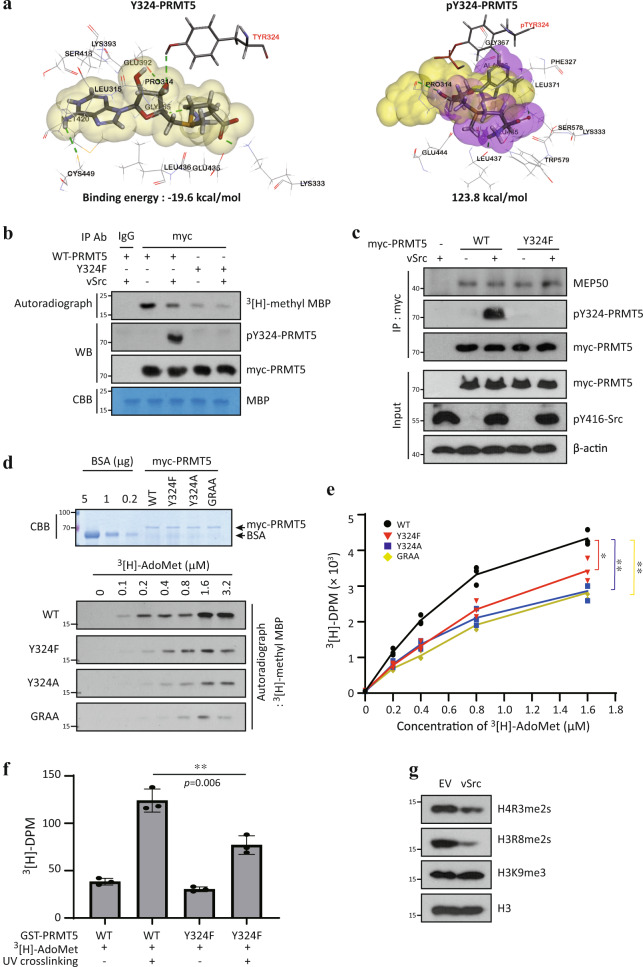
Y324 phosphorylation of PRMT5 suppresses its enzymatic activity. **a** Analysis of AdoMet interaction with active site of PRMT5. AdoMet interacts with intact PRMT5 through five hydrogen bonds including Y324 (left, yellow cloud). Upon phosphorylation of the Y324 (pTYR324) site, two hydrogen bonds are lost (right, purple cloud). **b** In vitro PRMT5 methyltransferase assay. Immunoprecipitated myc-PRMT5 from HEK293T cells were incubated with MBP and^3^[H]-AdoMet. Methylated samples were analyzed by SDS-PAGE and autoradiography. Amounts of myc-PRMT5 and MBP were evaluated by western blotting and Coomassie Brilliant Blue (CBB) staining, respectively. **c** Interaction of PRMT5 with MEP50. U2OS cells were co-transfected with myc-PRMT5 (WT or Y324F) and vSrc plasmids. Co-immunoprecipitated endogenous MEP50 is shown. **d**, **e** Analysis of PRMT5 enzymatic kinetics. Enzyme activities of myc-PRMT5 proteins (WT, Y324F, Y324A, or GRAA (G367A-R367A, enzymatic dead-from)) were measured by in vitro methyltransferase assays with increasing concentrations of ^3^[H]-AdoMet. Amount of myc-PRMT5 protein was evaluated by Coomassie Blue staining. Radioactivity of the ^3^[H]-labeled MBP protein was analyzed by autoradiography (**d**) and then quantitated by Liquid scintillation counter (**e**). Error bars represent standard deviation (SD, *n* = 3). *P*-value of WT vs Y324F: 0.015, WT vs Y324A: 0.0013, WT vs GRAA: <0.001. **f** UV-crosslinking assay of PRMT5 with AdoMet. Recombinant GST-PRMT5 (WT or Y324F) was purified from *E. coli* and then cross-linked with 3 μCi ^3^[H]-AdoMet using UV crosslinker. ^3^[H]-DPM count represents the amount of ^3^[H]-AdoMet cross-linked with GST-PRMT5 (*n* = 3). **g** Suppression of PRMT5-targeting histone arginine methylation by Y324 phosphorylation. U2OS cells were transfected vSrc plasmid for 2 days and then nuclear histone fraction was extracted. Equal amount of histone proteins were analyzed by western blotting. The autoradiographs in (**b**, **d**), immunoblots in (**b**, **c**, **g**) and CBB in (**b**, **d**) are representative of three independent experiments with similar results, respectively. *P < 0.05 and **P < 0.01.

Recent reports have shown that the Y324 residue is required for AdoMet-binding, as it mediates a hydrogen bond between PRMT5 and the AdoMet molecule^26,28^. In silico modeling of PRMT5 shows that Y324 residue is required for stabilization of AdoMet binding through the formation of a hydrogen bond with the ribose group of AdoMet (Fig. 2a, left). Importantly, Y324 phosphorylation likely leads to a loss of the hydrogen bond with AdoMet and a distortion of the structure of the AdoMet binding region (Fig. 2a, right). In addition, a comparison of binding energies reveals that un-modified PRMT5 and AdoMet are more tightly bound than phosphorylated Y324-PRMT5 and AdoMet (−19.6 and 123.8 kcal/mol, respectively). Thus, Y324 phosphorylation may influence PRMT5 enzyme activity. To further investigate this, we performed an in vitro methyltransferase assay using ^3^[H]-AdoMet. Myc-PRMT5 protein was purified from HEK293T cells co-transfected with empty vector or vSrc, and then incubated with myelin basic protein (MBP, a well-established substrate protein of PRMT5) and ^3^[H]-AdoMet. PRMT5 (co-expressed with an empty vector) strongly methylated MBP (Fig. 2b, lane 2); however, the MBP methylation was substantially suppressed by PRMT5 co-expressed with vSrc (Fig. 2b, lane 3), indicating that the methyltransferase activity of PRMT5 is suppressed by Src kinase-mediated phosphorylation. In addition, we observed that Y324F mutant has substantially lower enzyme activity, regardless of the co-expression with vSrc (Fig. 2b, lane 4 and 5). This was also verified by using another PRMT5 substrate, recombinant core histone proteins (Supplementary Fig. 1h). These results indicate that the integrity of the Y324 residue of PRMT5 is important for its enzyme activity, and that the Y324 phosphorylation might be a negative regulation mechanism for PRMT5 activity in cells. This was further strengthened by the observation that other mutants of Y324 (Y324A, Y324E, and Y324Q) have low enzyme activities similar to that of Y324F, which is as low as that of the GRAA mutant. (Supplementary Fig. 1i).

Next, we endeavored to clarify how PRMT5 activity is regulated by the Y324 phosphorylation. It has been well-documented that hetero-dimerization of PRMT5 with MEP50 is essential for PRMT5 activity^26^, and PRMT5-MEP50 heterodimer forms a tetramer complex^26,29^. However, the interaction between PRMT5 and MEP50 was not affected by Y324 phosphorylation or Y324F mutation (Fig. 2c). Next, we examined the degree of binding between PRMT5 and AdoMet, because the hydroxyl group of Y324 residue is required for the formation of hydrogen bond with AdoMet molecule and the stabilization of this binding (Fig. 2a). We first measured the kinetics of PRMT5 enzyme activity using various Y324 mutants. As the ^3^[H]-AdoMet concentrations increased, the wild type PRMT5 activity showed typical Michaelis–Menten kinetics with a Km of 0.42 ± 0.03 μM (Fig. 2d). In contrast, both Y324F and Y324A mutants displayed poor enzymatic activity with a Km of 0.75 ± 0.05 μM and 0.84 ± 0.02 μM, respectively (Fig. 2d). In addition, the UV-cross linking assay also showed that AdoMet binding capacity of Y324F mutant is clearly weak compared to wild type PRMT5 (Fig. 2e). Indeed, it was observed that the activity of intracellular PRMT5 was suppressed when vSrc was overexpressed (Fig. 2f). These results demonstrate that the Y324 residue of PRMT5 is indispensable for optimal AdoMet binding and enzymatic activity; phosphorylation or mutation of this residue interferes with AdoMet binding and consequently results in decreased methyltransferase activity.

### Y324 phosphorylation of PRMT5 occurs under DNA damage stress

**Fig. 3:**
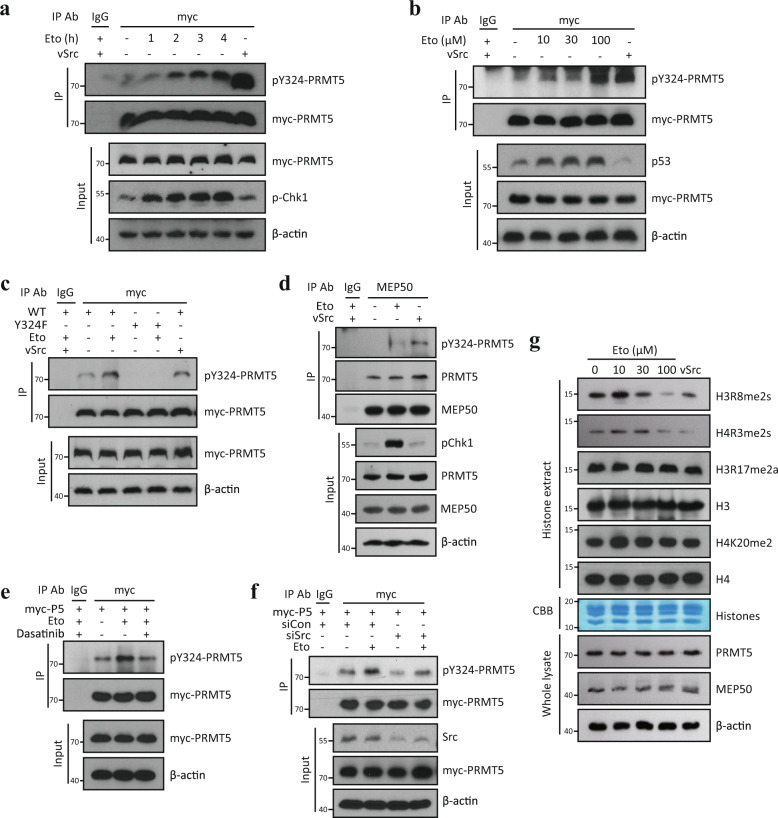
Y324 phosphorylation of PRMT5 occurs under DNA damage stress. **a**, **b** Y324 phosphorylation of PRMT5 under DNA damage stress. U2OS cells were transfected with myc-PRMT5 and then treated with 50 μM etoposide for indicated times (**a**) or indicated concentrations of etoposide for 2 h (**b**). Myc-PRMT5 was immunoprecipitated, followed by immunoblotting with pY324-PRMT5 antibody. vSrc kinase were used as positive control. **c** U2OS cells were transfected with WT or Y324F myc-PRMT5 and then treated with 50 μM etoposide for 2 h, followed by IP-western analysis. **d** Y324 phosphorylation of endogenous PRMT5 by etoposide. U2OS cells were treated with 50 μM etoposide for 2 h and then subjected to IP using MEP50 antibody. **e**, **f** Src kinase-dependent PRMT5 Y324 phosphorylation under DNA damage stress. e U2OS cells expressing myc-PRMT5 were pre-incubated with 1 μM dasatinib for 1 h, followed by treatment with 50 μM etoposide for 2 h. f U2OS cells were co-transfected with myc-PRMT5 and Src kinase-targeting duplex siRNA for 3 days, and then treated with 50 μM etoposide for 2 h. **g** Suppression of PRMT5-mediated histone methylation under DNA damage stress. U2OS cells were treated with indicated concentrations of etoposide for 24 h and then nuclear histone fraction was extracted. Equal amount of histone proteins were analyzed by western blotting. vSrc-transfected U2OS cells were used as positive control. The immunoblots in (**a**–**g**) and CBB in (**g**) are representative of three independent experiments with similar results, respectively.

Src kinase has been implicated in many cellular processes, including cell proliferation and survival pathways under growth factor signals^30,31^. To determine what cellular process might signal the Src kinase to phosphorylate the Y324 residue, we first investigated the effects of growth factor stimulation on this phosphorylation event. Under EGF or PDGF stimulation, Y324 phosphorylation of PRMT5 was not induced (Supplementary Fig. 2a and b). We next determined whether the Y324 phosphorylation occurs in response to DNA damage, because several reports have demonstrated the role of PRMT5 in DNA damage responses^9,10,12,15,32^. Importantly, under etoposide-induced DNA damage stresses, the phosphorylation of the Y324 residue increased in a time- and dose-dependent manner (Fig. 3a and b), and was completely abolished in the Y324F mutant (Fig. 3c). Endogenous PRMT5 was also phosphorylated by etoposide (Fig. 3d). In addition, the Y324 phosphorylation was substantially blocked by dasatinib or Src kinase depletion (Fig. 3e and f), supporting a central role for Src kinase in mediating Y324 phosphorylation under DNA damage stress. We found that the Y324 phosphorylation of PRMT5 occurred not only by etoposide (topoisomerase II inhibitor) but also by camptothecin (CPT, topoisomerase I inhibitor), doxorubicin (DNA intercalator) and UVB irradiation (formation of thymine dimers or other pyrimidine dimers and double-strand DNA breakage) (Supplementary Fig. 2c–f), indicating that PRMT5 enzyme activity is suppressed under wide diversity of DNA lesions. Indeed, the levels of PRMT5-mediated methylation mark, H3R8me2s, and H4R3me2s were dramatically reduced under DNA damage stress without affecting PRMT5-independent H3R17me2a or H4K20me2 levels (Fig. 3g). We ruled out the possibility of PDGFRβ-mediated Y324 phosphorylation in DNA damage responses because PDGFRβ depletion did not affect the etoposide-induced PRMT5 Y324 phosphorylation (Supplementary Fig. 2g). These results indicate that PRMT5 activity is inhibited by Src kinase-mediated Y324 phosphorylation during cellular responses to DNA damage.

### PRMT5 regulates 53BP1 stability by methylating its GAR motif

In order to establish the biological role of PRMT5 Y324 phosphorylation in DNA damage responses, we first examined DNA damage-induced CHK1 and CHK2 signaling pathways. Depletion of PRMT5 diminished CHK1 phosphorylation (p-Ser345) and slightly reduced CHK2 phosphorylation (p-Thr68, Supplementary Fig. 3a). Consistent with our data, it has been reported that PRMT5 methylates Rad9, a key adaptor protein in DNA damage checkpoint pathways, and the activation of the Rad9 downstream checkpoint effector CHK1 is impaired in cells expressing a arginine methylation-deficient mutant Rad9^10^. However, we found that overexpression of wild type or Y324F PRMT5 did not alter the levels of phosphorylated CHK1 or CHK2 (Supplementary Fig. 3b). The discrepancy between these depletion and mutant overexpression experiments seems to be probably due to differences in the net activity of PRMT5 throughout the cells. Rather, γH2AX level was negatively correlated with PRMT5 activity (Supplementary Fig. 3a and b), implying that PRMT5 activity may be involved in sensitivity or restoration for DNA damage.

**Fig. 4:**
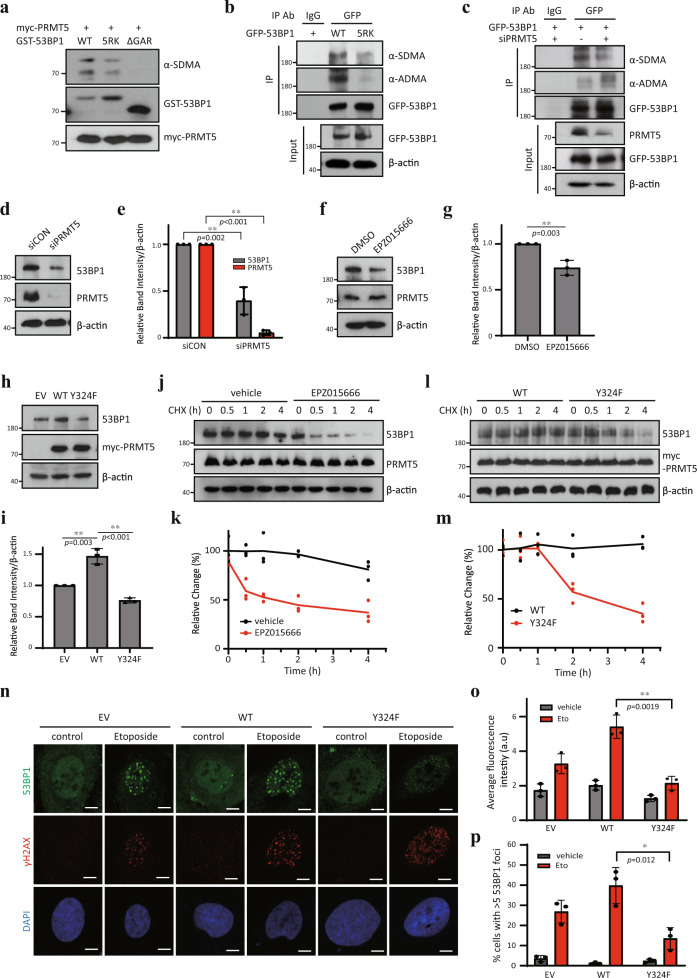
53BP1 stability is controlled by PRMT5. **a** In vitro methylation of 53BP1 by PRMT5. Recombinant GST-53BP1-Tudor (focus forming region containing tandem Tudor domains, aa 1220–1711)-wild type (WT), −5RK, and −∆GAR were incubated with myc-PRMT5 purified from HEK293T and 10 μM cold AdoMet 37 °C for 1 h in vitro. Samples were analyzed by immunoblotting. **b**, **c** Symmetric dimethylation of GAR motif in 53BP1 by PRMT5. HEK293T cells were transfected with WT or 5RK GFP-53BP1 (**b**) or co-transfected with GFP-53BP1 and siPRMT5 (**c**), and then subjected to immunoprecipitation using GFP antibody. **d**–**i** Regulation of 53BP1 protein level by PRMT5. U2OS cells were transfected with siPRMT5 for 3 days (**d**, **e**), treated with 10 μM EPZ015666 for 2 days (**f**, **g**) or overexpressed with WT or Y324F myc-PRMT5 for 2 days (**h**, **i**). The intensity of protein bands was quantified using image processing software (*n* = 3). **j**–**m** Analysis of 53BP1 protein stability. (**j**, **k**) U2OS cells were treated with DMSO or 10 μM EPZ015666 for 16 h, and then treated with 50 μg/ml cycloheximide for the indicated times. **l**, **m** U2OS cells were transfected with WT or Y324F myc-PRMT5 for 1 day, and then co-treated with 50 μg/ml cycloheximide for the indicated times. Band intensities were quantitated using image processing software. Error bars indicate standard deviation of 3 independent replicates. **n**–**p** IF Analysis of 53BP1 foci formation. U2OS cells overexpressing empty vector (EV), WT or Y324F myc-PRMT5 were treated with 10 μM etoposide for 2 h and then assessed by co-immunostaining for 53BP1 (green) and γH2AX (red), followed by DAPI staining. **n** Representative pictures are shown. Scale bar: 5 μm. **o** The fluorescence intensity of 53BP1 was quantified by image analysis software (*n* = 3). **p** Quantification of the percentage of cells with ≥5 53BP1 foci (*n* = 3). *P < 0.05 and **P < 0.01. The immunoblots in (**a**–**d**, **f**, **h**, **j**, **l**) are representative of three independent experiments with similar results, respectively.

Next, we tried to find selective substrate protein(s) regulated by PRMT5 in DNA damage responses. 53BP1 protein is a key regulator of non-homologous end joining (NHEJ) DNA repair process, which has a glycine-arginine rich (GAR) motif that is methylated by PRMT1^33^. We have found that GAR motif of 53BP1 was symmetrically dimethylated by PRMT5 in vitro (Fig. 4a and Supplementary Fig. 4a), which was confirmed by the observations that both SDMA and ADMA were clearly detected in wild type 53BP1 but not in 5RK (a mutant in which all five arginine residues within GAR motif are substituted to lysines, Fig. 4b). Moreover, SDMA was reduced and ADMA was profoundly increased upon PRMT5 depletion or Y324F overexpression (Fig. 4c and Supplementary Fig. 4b), however, PRMT1 depletion showed reversed results (Supplementary Fig. 4c), indicating a possible competition for SDMA and ADMA on 53BP1 by PRMT5 and PRMT1. Interestingly, we observed that 53BP1 protein level gets substantially downregulated by depletion or inhibition of PRMT5 (Fig. 4d and e). This was further verified in different sets of *PRMT5* siRNA- or shRNA-transfected cells (Supplementary Fig. 4c and d). In addition, overexpression of wild type PRMT5, but not Y324F mutant, led to an increase in 53BP1 protein levels (Fig. 4f). The mRNA level of *53BP1* was not influenced by either overexpression or depletion of PRMT5 (Supplementary Fig. 4e and f), suggesting that PRMT5 regulates the post-transcriptional levels of 53BP1.

Because there are several evidence for protein stability being regulated by arginine methylation^9,32,34^, we investigated whether the protein stability of 53BP1 is controlled by PRMT5. Upon treatment with cycloheximide, the levels of 53BP1 protein in resting condition were rapidly diminished in PRMT5 inhibitor-treated or Y324F-expressing cells without affecting cell viability or cell cycle progression (Fig. 4g and h; Supplementary Fig. 5a and b). De-stabilization of 5RK-53BP1 was also detected (Supplementary Fig. 5e). The turnover of 53BP1 is accelerated by DNA damage^35^, we even confirmed that protein stability of 53BP1 under etoposide treatment is also regulated by PRMT5 activity (Supplementary Fig. 5f). Consistent with these data, the fluorescence intensity of 53BP1 was considerably weakened in PRMT5-depleted or Y324F-expressing cells compared to the control (Supplementary Fig. 5g, Fig. 4i right upper). Furthermore, while 53BP1 was intensively recruited to nuclear foci in wild type PRMT5 or scrambled siRNA-transfected cells, both Y324F mutant and siPRMT5-transfected cells showed weak intensity in 53BP1 foci (Fig. 4i right lower; Supplementary Fig. 5g). Recent studies show that PRMT5 depletion or knockout enhanced 53BP1 foci upon IR^36,37^. Similar to these results, we also observed that etoposide-induced 53BP1 foci was formed in PRMT5-depleting or Y324F- expressing cells. However, the overall intensity and the number of high intensity 53BP1 foci were significantly decreased. Collectively, these data suggest that PRMT5 symmetrically dimethylates the GAR motif in 53BP1, a protein that is tightly associated with NHEJ repair processes, leading to an increase in its stability.

### PRMT5 contributes to NHEJ and cell survival after DNA damage

**Fig. 5:**
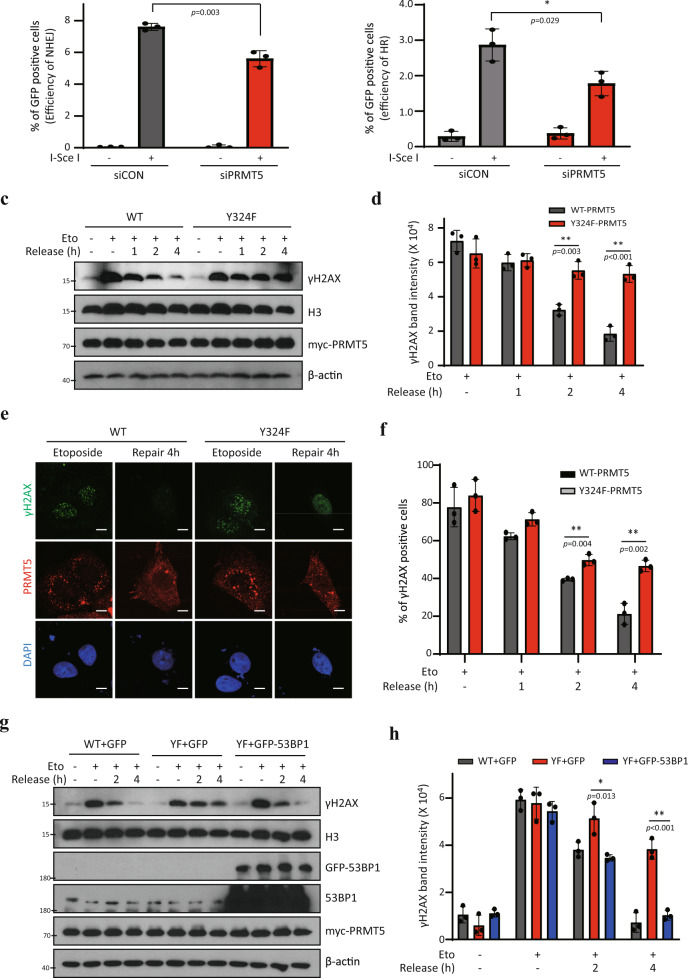
PRMT5 activity is required for DNA repair process and cell survival after DNA damage. **a**, **b** NHEJ or HR reporter assays in PRMT5-depleted cells. U2OS stable cell lines expressing either EJ5-GFP (**a**) or DR-GFP (**b**) reporter constructs were co-transfected with PRMT5 siRNA and I-SceI plasmids. After 3 days, the efficiency of NHEJ (**a**) or HR (**b**) was determined by counting the number of GFP-positive cells using flow cytometry (*n* = 3). **c**–**f** Analysis of γH2AX levels during DNA repair process. U2OS cells overexpressing myc-PRMT5 (WT or Y324F) were treated with 10 μM etoposide for 2 h, and then incubated with fresh media for the times indicated. Cells were harvested and subjected to immunoblotting (**c**, **d**) or immunostaining (**e**, **f**) analysis. c Representative western bands of three independent experiments are shown. **d** The γH2AX band intensity in (**c**) was quantified by image processing software (*n* = 3). **e** Representative pictures are shown. Scale bar: 5 μm. **f** The percentage of γH2AX positive cells in (**e**) was quantified (*n* = 3). **g**, **h** U2OS cells were co-transfected with myc-PRMT5 and GFP-53BP1 as described, and then exposed to 10 μM etoposide for 2 h, followed by incubation with fresh media for indicated times. **g** The representative western bands of three independent experiments are shown. **h** The γH2AX band intensity in (**g**) was quantified by image processing software (*n* = 3). *P < 0.05 and **P < 0.01.

Because 53BP1 is a critical regulator of the DNA repair process, especially NHEJ pathway^22,24,38,39^, we further characterized the role of PRMT5 activity in DNA repair processes using EJ5-GFP (NHEJ) and DR-GFP (homologous recombination, HR) reporter assays^40,41^ (Supplementary Fig. 6a and b). We detected that PRMT5 depletion resulted in a significant reduction of both NHEJ and HR-dependent repair process (Fig. 5a and b; Supplementary Fig. 6c and d). Consistent with our data, recent study has been reported that PRMT5 is involved in HR pathway by methylating the TIP60 cofactor RUVBL1^37^. Here, we identified that PRMT5 is obviously involved in NHEJ repair process as well as HR, presumably through the regulation of 53BP1 stability. In addition, etoposide-induced γH2AX levels rapidly decrease after replacement with fresh media; however, depletion of endogenous PRMT5 resulted in sustained γH2AX levels (Supplementary Fig. 6e). Furthermore, reduced dephosphorylation of γH2AX was also observed in Y324F-overexpressing cells (Fig. 5c), indicating that PRMT5 activity is required for efficient DNA repair. To confirm this, we analyzed the nuclear γH2AX foci formation using confocal microscopy. Importantly, γH2AX foci were sustained for a longer time in PRMT5-depleted or Y324F-overexpressing cells (Fig. 5d, Supplementary Figs. 6f, and  7a). The level of H4K20me2, known to be involved in the recruitment of 53BP1 to the DNA foci^22,42^, was not changed during the DNA repair process (Supplementary Fig. 7b), which is consistent with the previous observation in Fig. 3f. The retardation in the recovery of γH2AX in the Y324F mutant was rescued by overexpression of GFP-53BP1 (Fig. 5e), confirming that the role of PRMT5 in DNA damage repair process is mediated by 53BP1.

**Fig. 6:**
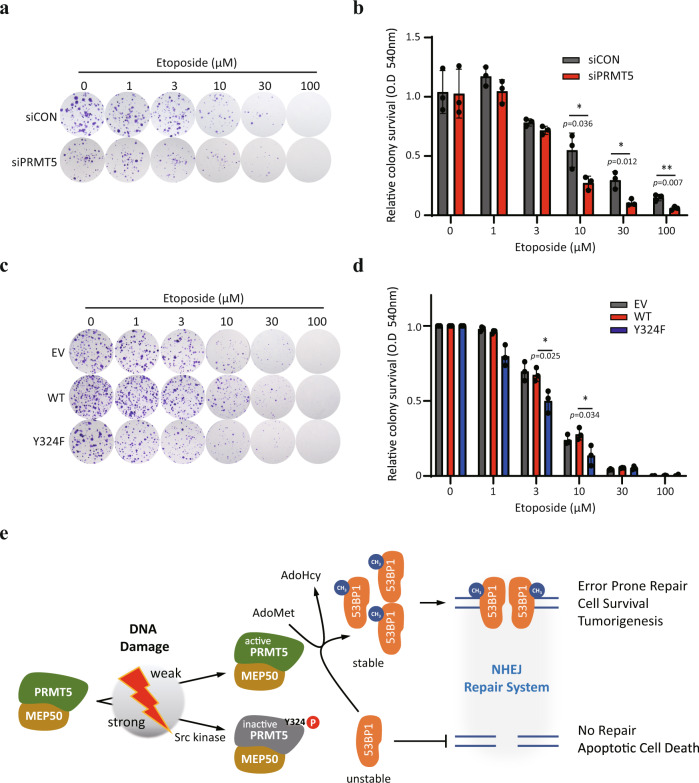
PRMT5 regulates cell survival after DNA damage. **a**–**d** MCF7 cells were transfected with siRNA (**a**, **b**) or expression vector (**c**, **d**) as described, and then exposed to etoposide for 2 h. After etoposide exposure, cells were incubated with fresh media for 2 weeks. Cell colonies were stained with Crystal violet. **a**, **c** Representative picture of colonies from three independent experiments. **b**, **d** Analysis of colony-forming rate by measurement of absorbance at 540 nm. Data are means ± SD of three independent experiments (*P < 0.05 and **P < 0.01). **e** A model for the regulation of PRMT5 activity. PRMT5 activity may act as a major determinant of whether the cells undergo DNA repair or apoptosis in response to DNA damage.

To further strengthen the role of PRMT5 in DNA repair process, we examined whether cell survival rates could be governed by PRMT5 levels/activity, after treatment with a DNA damaging agent. Cell survival rate after treatment with etoposide significantly decreased in either PRMT5-depleted or Y324F-PRMT5 overexpression cells (Fig. 6a and b). Taken together, these data strongly suggest that PRMT5 is a critical player in cell recovery from DNA damage and implicate it in the DNA repair pathway, in part, by stabilization of 53BP1 protein (Fig. 6c).

## Discussion

Our studies have revealed a molecular mechanism by which Y324 phosphorylation of PRMT5 by Src kinase negatively regulates its methyltransferase activity by impeding AdoMet binding. In addition, this phosphorylation and inhibition of PRMT5 occurs during DNA damage response. We also demonstrated that PRMT5 is a critical regulator for the recovery of a cell from DNA damage, and this function lies in both HR and NHEJ repair pathway. These findings raise the fundamental question of why PRMT5 activity is suppressed via Y324 phosphorylation during the repair of damaged DNA. Since PRMT5 activity is involved in DNA repair process, if the cells are subjected to minor DNA damage, PRMT5 activity may be maintained to recover from the damage. However, if DNA is severely damaged, it may be necessary to block the repair process by inhibiting PRMT5 activity and inducing apoptosis. Thus, it is possible that the regulation of PRMT5 activity may act as a major determinant of whether the cells undergo DNA repair or apoptosis in response to DNA damage. Essentially, PRMT5 can somehow gauge the degree of DNA damage that a cell has sustained (Fig. 6c). This idea is supported by the observation that Y324 phosphorylation of PRMT5 is observed only at high concentrations of a DNA damage agent (Fig. 3b), which is enough to induce apoptosis (Supplementary Fig. 8a and b).

In many human cancers such as liver, breast, cervix, prostate, lung, and colon cancer, PRMT5 is highly expressed and associated with their poor prognosis^3,16,43–45^. In addition, elevated PRMT5 expression in both breast cancer and lung cancer correlates with poor survival (http://www.kmplot.com, Supplementary Fig. 8c and d). In this study, our studies describe a possible mechanism, at least in part, underlying these oncogenic functions of PRMT5; the symmetric dimethylation of 53BP1 by PRMT5 results in stabilization of its protein level and promotion of NHEJ efficiency in cancer cells. Because NHEJ repair is known to be an error-prone pathway^38,46^, genomic instability might be increased in PRMT5-overexpressing cells, leading to a pro-growth tumor phenotype. In addition, 53BP1 is correlated to cancer cell survival after DNA damaging treatment^21,47,48^, the stabilization of 53BP1 by PRMT5 might contribute to radiotherapy resistance of cancer cells. Therefore, overexpression of PRMT5 would be a remarkable biomarker of poor prognosis and a good target for cancer treatment.

53BP1 is a key regulator of NHEJ repair processes by forming large foci near DNA lesions where ATM- or ATR-mediated DNA damage signaling is induced^21,22,39^. 53BP1 contains several functional domains for numerous NHEJ-responsive proteins including the BRCA1 carboxy-terminal (BRCT) repeats, the tandem Tudor domains, an oligomerization domain (OD), a GAR motif, an ubiquitination-dependent recruitment (UDR) motif, and 28 amino-terminal Ser/Thr-Gln (S/T-Q) sites^21,22^. It has been reported that the GAR motif is asymmetrically dimethylated by PRMT1 and this methylation is required for DNA binding affinity of 53BP1 rather than its oligomerization^33,49^. However, this notion has been challenged by other observations that the localization of 53BP1 into foci is driven by physical interaction between the tandem Tudor domain of 53BP1 and lysine-methylated histones, not by asymmetric dimethylation of the GAR motif^50–52^. In this study, we show that the GAR motif can also be symmetrically dimethylated by PRMT5, which leads to an increase in stability of 53BP1. It has been reported that ubiquitin-conjugating enzyme H7 (UbcH7, an ubiquitin E2 enzyme)^53^, ring finger protein RNF8/RNF168 (both E3 ligases)^35^ and Nudix hydrolase NUDT16^54^ are involved in proteosomal degradation of 53BP1, respectively. Nevertheless, the mechanisms that regulate protein stability of 53BP1 are still not well understood. Further studies are needed to clarify the correlation between PRMT5-mediated arginine methylation and RNF8/RNF168-mediated ubiquitination or NUDT16-mediated ADP-ribosylation. Because accumulating evidence support that upregulation of 53BP1 enhances mutagenic NHEJ repair and radioresistance, which is correlated with reduced survival rate and poor prognosis in several tumor^55,56^, a precise regulation of 53BP1 levels is critical for tumor development and therapeutic outcome^57^. In this point of view, our proposed mechanisms on 53BP1 stabilization could provide valuable information for cancer prevention and treatment.

Collectively, our findings report a previously unidentified regulatory mechanism for PRMT5 methyltransferase activity through Src kinase-induced Y324 phosphorylation, and the involvement of PRMT5 in NHEJ repair pathway through regulation of 53BP1 stability, which can be targeted for cancer treatment.

## Methods

### Constructs, reagents, and antibodies

Myc-PRMT5 plasmids were obtained from M.T. Bedford and were sub-cloned into a pcDNA3 vector containing N-terminal FLAG-tag and a pGEX vector containing N-terminal glutathione S-transferases (GST) tag for the purification of recombinant protein. PRMT5 Y324F, Y324A, Y324E, Y324Q, Y324S, Y324T, and PRMT5-G367A-R368A mutants were generated using the Muta-direct^TM^ site-directed Mutagenesis kit (iNtRON BIOTECHNOLOGY, Seongnam, Korea) according to the manufacturer’s protocol. Plasmid containing GFP tagged-human 53BP1 was purchased from Origene (Rockville, MD) and then subjected to site-directed mutagenesis to generate an arginine methylation-deficient mutant (5RK, five Arg to Lys starting from Arg 1396 to Arg 1403 (R1396, R1398, R1400, R1401 and R1403) in GAR motif). GST-53BP1 deletion mutants (GST-53BP1-Tudor (1220–1711) -WT, -5RK, -∆GAR (deletion from 1381 to 1480) were cloned using pGEX vector and GFP-53BP1 plasmid. Etoposide and camptothecin were obtained from Enzo Life Sciences (Farmingdale, NY). EGF, dasatinib, and doxorubicin were obtained from Sigma-Aldrich (St. Louis, MO), while PDGF-AA was from PROSPEC (Rehovot, Israel). The following peptides were chemically synthesized by AbClone (Seoul, Korea) for antibody production in rabbit and dot blot assays: Unmodified peptide (NH_2_-PLMDNLESQTYEVFEKDPIKY-COOH) and phosphorylated peptide (NH_2_-PLMDNLESQT(pY)EVFEKDPIKY-COOH). Antibodies against pY100 (1:5000), pY416-Src kinase (1:10,000), γH2AX (1:10,000), histone H3 (1:10,000), H4 (1:5000), p-CHK1 (1:5000), CHK1 (1:5000), p-CHK2 (1:5000), CHK2 (1:2000), and cleaved caspase-3 (1:1000) were purchased from Cell Signaling Technology (Danvers, MA). Anti-PRMT5 (1:5000), anti-cSrc kinase (1:5000), anti-SDMA (Sym10, 1:5000), and anti-ADMA (Asym24, 1:5000) were purchased from Millipore (Billerica, MA). Antibodies against c-myc (1:10,000), β-actin (1:10,000), and PARP (1:10,000) were from Santa Cruz (Dallas, TX). Anti-GFP (,1:10,000) was purchased from Invitrogen (Carlsbad, CA), and anti-53BP1 (1:10,000), anti-MEP50 (1:10,000), Alexa594-conjugated goat anti-mouse (1:2000), and FITC-conjugated goat anti-rabbit (1:2000) secondary antibodies were purchased from Bethyl Laboratories (Montgomery, TX). Anti-H3R8me2s (1:10,000), anti-H4R3me2s (1:10,000), anti-H3R17me2a (1:10,000) and anti-H4K20me2 (1:10,000) were from Abcam (Cambridge, UK). HRP-conjugated secondary antibodies (1:100,000) were purchased from Jackson ImmunoResearch Laboratories (West Grove, PA).

### Cell culture and transfection

HEK293T (Human Embryonic Kidney 293T cells), U2OS (human osteosarcoma cells), MCF7 (human breast cancer cells), A549 (human non-small cell lung cancer cells), and NIH3T3 (mouse fibroblast cells) were obtained from American Type Culture Collection (ATCC, Manassas, VA). All cell lines were grown in Dulbecco’s modified Eagle’s medium (DMEM) supplemented with 10% heat-inactivated fetal bovine serum (FBS; HyClone Laboratories, Logan, UT) and 100 units/ml penicillin-streptomycin (HyClone Laboratories). Cells were maintained at 37 °C under 5% CO_2_ in a humidified chamber. For siRNA transfection, cells were transfected with TransIT-X2 (Mirus Bio, Madison, WI) according to the manufacturerʼs instructions. All siRNA sequences were synthesized by IDT (Integral DNA Technologies, Singapore) and all experiments were performed at least 2 days after knockdown. For overexpression from ectopic mammalian expression plasmids, cells were transfected with TransIT-2020 (Mirus Bio).

### Immunoblotting and immunoprecipitation

Whole-cell extracts were obtained using lysis buffer (20 mM Tris-HCl pH 8.0, 150 mM NaCl, 10% glycerol, 1% NP40, 2 mM EDTA) supplemented with 1X protease and phosphatase inhibitor cocktails (Roche, Basel, Switzerland). Cell extracts were incubated for 20 min in an ice bath, followed by sonication and then centrifuged to clarify lysates. Protein concentration was determined by Bradford Assay according to the manufacturer’s instructions (BioRad, Hercules, CA). After boiling in Laemmli sample buffer for 5 min, equal amounts of protein lysates were resolved by SDS-PAGE, and then transferred onto PVDF. The membranes were blocked with 5% skim milk/0.1% Tween 20/TBS (Tris-buffered saline) for 1 h at room temperature and incubated with primary antibody overnight, followed by HRP-linked secondary antibody for 1 h at room temperature. The signal was detected using ECL western blotting substrate (Advansta, Menlo Park, CA). The band intensity of western band was analyzed by Image Studio version 5.0 (LI-COR Biotechnology, Lincoln, NE).

For preparation of lysates for immunoprecipitation (IP), cells were washed three times in ice-cold phosphate-buffered saline (PBS) and lysed with NP40 lysis buffer (20 mM Tris-HCl pH 7.5, 150 mM NaCl, 10% Glycerol, 1% NP40, 2 mM EDTA) supplemented with 1X protease and phosphatase inhibitor cocktails. After centrifugation, appropriate antibodies were added at a concentration of 1 mg/ml of lysate and incubated overnight at 4 °C, followed by antibody-protein complex capture using Protein A/G Sepharose beads (SantaCruz, Dallas, TX) for at least 1 h at 4 °C. After washing twice with NP40 lysis buffer, the complexes were eluted and analyzed by SDS-PAGE and immunoblotting.

### Immunofluorescence, confocal microscopy, and image analysis

Cells were plated on coverslips for 24 h prior to transfection with siRNAs or DNA plasmids. After experimental treatment, cells were fixed for 30 min in 4% PFA, washed with PBS three times, and permeabilized and blocked with 0.25% Triton X-100 and 10% BSA in PBS for 1 h. Cells were then incubated with primary antibody overnight at 4 °C and with fluorescence-conjugated secondary antibody for 1 h at room temperature. After DAPI (Thermo Fisher Scientific, Waltham, MA) staining for 5 min, cells were mounted onto glass slides. Staining was assessed using a Zeiss LSM 710 immunofluorescence microscope and ZEN software (Carl Zeiss, Oberkochen, Germany). Cells with ≥5 foci per cell were classified as foci-positive. A minimum of 300 cells were counted for each experimental repeat. Representative images were obtained at ×100 magnification. The fluorescence intensity of pictures was analyzed by Image Studio version 5.0. Antibodies used include γH2AX (Cell Signaling Technology, #2577, 1:2000), 53BP1 (Bethyl Laboratories, A300-272, 1:2000), and PRMT5 (Millipore, 07–405, 1:1000).

### Quantitative real-time PCR

Total cellular RNA was extracted using TRIsure^TM^ (Bioline, London, UK) and cDNA synthesized using the SensiFAST^TM^ cDNA synthesis kit (Bioline). The synthesized cDNA was used as template for real-time PCR which was performed using the Eco Real-Time PCR System (Illumina, San Diego, CA) and the SensiFAST SYBRTM No-ROX Kit (Bioline). Reaction parameters were as follows: cDNA synthesis at 37 °C for 60 min, transcriptase inactivation at 95 °C for 5 min, PCR cycling at 95 °C for 10 s, 58 °C for 30 s, and 72 °C for 20 s, for 40 cycles. The primer sets for PRMT5 were 5′-TTTCCCATCCTCTTCCCTATTAAG-3′ and 5′-CCCACTCATACCACACCTTC-3′; the primer sets for 53BP1 were 5′-GGCTACGCATTTCTCCTTACC-3′ and 5′-AAGCTGGGATTCTGTATACTGC-3′.

### In vitro Src kinase assay

In vitro Src kinase assay was performed using immunoprecipitated Src kinase from vSrc-transfected A549 cells, purified myc-PRMT5 from HEK293T cells, and 1 mM ATP in kinase buffer (25 mM Tris-HCl (pH 7.5), 150 mM NaCl, 2 mM DTT, 0.1 mM Na_3_VO_4_, 10 mM MgCl_2_) supplemented with 1X protease and phosphatase inhibitor cocktail. Reactions were incubated at 37 °C for 1 h, and proteins were then resolved by gel electrophoresis.

### In vitro methylation assay

Myc-PRMT5 was purified from transfected HEK293T cells by anti-myc immunoprecipitation. Immobilized myc-PRMT5 proteins were then incubated with 50 μl of reaction buffer (20 mM Tris-HCl (pH 7.5), 150 mM NaCl, 2 mM EDTA, 1 mM PMSF, 1 mM DTT) supplemented with 1 μg of recombinant histone (mixture of H2A, H2B, H3, and H4; New England Biolabs, Ipswich, MA) or 1 μg MBP (myelin basic protein; Millipore) and 1 μCi ^3^[H]-AdoMet (specific activity: 55–85 Ci/mmol; PerkinElmer, Waltham, MA) at 37 °C for 1 h. The reaction was stopped by adding SDS loading buffer, and the proteins were resolved on SDS-PAGE gels. Proteins were transferred onto PVDF membranes, and the tritium signal was amplified by treating membranes with EN3HANCE (PerkinElmer) spray. Membranes were exposed to autoradiography film for at least 1 week at 80 °C.

### Calf intestinal phosphatase (CIP) assay

HEK293T cells were co-transfected with myc-PRMT5 and vSrc plasmid for 2 days, and then myc-PRMT5 proteins were immunoprecipitated with anti-myc antibody. Immunoprecipitated PRMT5 was used in CIP assay (New England Biosciences) according to the manufacturerʼs method. In brief, beads were incubated with or without 1 unit of CIP in NEB buffer at 37 °C for 60 min, and the reaction was stopped by adding SDS loading buffer. The samples were analyzed by SDS-PAGE and western blotting.

### HR and NHEJ assays

U2OS stable cell lines harboring DR-GFP and EJ5-GFP construct were kind gifts from Prof. Yonghwan Kim (Sookmyung Women’s University, Seoul, Korea). U2OS-DR-GFP or U2OS-EJ5-GFP cells were seeded into six-well plates and co-transfected with 1 μg of I-SceI vector and the indicated siRNA or DNA plasmid, using TransIT-2020 (Mirus Bio). After three days, cells were harvested by trypsinization and washed with PBS. The GFP signal arising from the recombination event was measured by flow cytometry (FACSCalibur, BD Biosciences, Franklin Lakes, NJ). Fluorescence was detected in the FL1-H channel (logarithmic scale). Each data point represents the average ± standard deviation from three independent experiments.

### UV cross-linking assay

One microgram of GST-PRMT5, 3 μCi ^3^[H]-AdoMet, and 1 mM DTT were mixed in 1× PBS and exposed to UV light (254 nm, 150 mJ/cm^2^) at a distance of 1 cm for 30 min at 4 °C using UVGL-58 UV cross-linker (UVP, Upland, CA). After cross-linking, samples were boiled with Laemmli sample buffer, separated on 7% SDS/PAGE, transferred to PVDF membrane, Coomassie-stained, destained, and dried. The amount of cross-linked ^3^[H]-labeled GST-PRMT5 was quantified by Liquid scintillation counting.

### Colony survival assay

MCF7 cells were plated at low density 24 h prior to transfection with siCON, siPRMT5, empty vector, WT-, or Y324F-FLAG PRMT5. Cells were treated with 10 μM etoposide for 2 h, followed by supplementation with fresh complete media. After 14 days, colonies were fixed with 4% PFA and stained with 0.05% crystal violet. Stained cells were washed three times with deionized water and then entirely dried. Data were normalized to respective non-treatment control.

### Molecular docking analysis

The molecular docking analysis was done using Discovery Studio 2016 software (Accelrys, San Diego, CA) and implementing the CHARMM force field. X-ray crystal structure of PRMT5 (Protein Data Bank code 4GQB) was obtained from the RCSB protein databank, and phospho-Y324 PRMT5 was modified from the structure of the WT form of PRMT5. The protein structures of WT and phospho-Y324 PRMT5 were optimized by smart minimizer algorithm to the energy minimized state. To dock the ligands, the Ligandfit docking method was used. The parameters of Ligandfit were validated using ligands from the PRMT5 crystal structure. The crystallographic ligand (2S,5S,6E)-2,5-diamino-6-[(3S,4R,5R)-5-(6-amino-9H-purin-9-yl)-3,4-dihydroxydihydrofuran-2(3H)-ylidene]hexanoic acid (PDB ID: 0XU) was used to determine the binding site. AdoMet was docked to the binding site of both proteins and 30 poses were generated for each. Based on the docking results, various scoring functions (Ligscore1_Dreiding, Ligscore2_Dreiding, PLP1, PLP2, PMF, DOCKSCORE) were determined and used to calculate the predicted binding energies.

### Statistics and reproducibility

The data are presented as mean ± SD from three independent experiments. Comparison between data from two groups was performed using a *t*-test for independent samples and *P*-value < 0.05 were considered statistically significant. *P < 0.05 and **P < 0.01. For experiments that are difficult to analyze statistically (e.g., immunoblotting and immunoprecipitation), they were repeated at least three times. The exact number of replicates are presented in individual figure legends.

### Reporting summary

Further information on research design is available in the Nature Research Reporting Summary linked to this article.

## Supplementary information

Supplementary figure 1

Supplementary Data 1

Supplementary information

Reporting Summary

Peer review

## Data Availability

The data that support the findings of this study are available from the corresponding author (Y.K.K.) upon reasonable request.

## References

[1] Bedford MT (2007). Arginine methylation at a glance. J. Cell Sci..

[2] Blanc RS, Richard S (2017). Arginine methylation: the coming of age. Mol. Cell.

[3] Yang Y, Bedford MT (2013). Protein arginine methyltransferases and cancer. Nat. Rev. Cancer.

[4] Dacwag CS, Ohkawa Y, Pal S, Sif S, Imbalzano AN (2007). The protein arginine methyltransferase Prmt5 is required for myogenesis because it facilitates ATP-dependent chromatin remodeling. Mol. Cell Biol..

[5] Fan H (2014). SKB1/PRMT5-mediated histone H4R3 dimethylation of Ib subgroup bHLH genes negatively regulates iron homeostasis in *Arabidopsis thaliana*. Plant J..

[6] Tae S (2011). Bromodomain protein 7 interacts with PRMT5 and PRC2, and is involved in transcriptional repression of their target genes. Nucleic Acids Res.

[7] Andreu-Perez P (2011). Protein arginine methyltransferase 5 regulates ERK1/2 signal transduction amplitude and cell fate through CRAF. Sci. Signal..

[8] Bezzi M (2013). Regulation of constitutive and alternative splicing by PRMT5 reveals a role for Mdm4 pre-mRNA in sensing defects in the spliceosomal machinery. Genes Dev..

[9] Cho EC (2012). Arginine methylation controls growth regulation by E2F-1. EMBO J..

[10] He W (2011). A role for the arginine methylation of Rad9 in checkpoint control and cellular sensitivity to DNA damage. Nucleic Acids Res..

[11] Hsu JM (2011). Crosstalk between Arg 1175 methylation and Tyr 1173 phosphorylation negatively modulates EGFR-mediated ERK activation. Nat. Cell Biol..

[12] Jansson M (2008). Arginine methylation regulates the p53 response. Nat. Cell Biol..

[13] Kirino Y (2009). Arginine methylation of Piwi proteins catalysed by dPRMT5 is required for Ago3 and Aub stability. Nat. Cell Biol..

[14] Reintjes A (2016). Asymmetric arginine dimethylation of RelA provides a repressive mark to modulate TNFalpha/NF-kappaB response. Proc. Natl Acad. Sci. USA.

[15] Zheng S (2013). Arginine methylation-dependent reader-writer interplay governs growth control by E2F-1. Mol. Cell.

[16] Stopa N, Krebs JE, Shechter D (2015). The PRMT5 arginine methyltransferase: many roles in development, cancer and beyond. Cell Mol. Life Sci..

[17] Liu F (2011). JAK2V617F-mediated phosphorylation of PRMT5 downregulates its methyltransferase activity and promotes myeloproliferation. Cancer Cell.

[18] Jackson SP (2002). Sensing and repairing DNA double-strand breaks. Carcinogenesis.

[19] van Gent DC, Hoeijmakers JH, Kanaar R (2001). Chromosomal stability and the DNA double-stranded break connection. Nat. Rev. Genet..

[20] Subba Rao K (2007). Mechanisms of disease: DNA repair defects and neurological disease. Nat. Clin. Pract. Neurol..

[21] Panier S, Boulton SJ (2014). Double-strand break repair: 53BP1 comes into focus. Nat. Rev. Mol. Cell Biol..

[22] Zimmermann M, de Lange T (2014). 53BP1: pro choice in DNA repair. Trends Cell Biol..

[23] Gaymes TJ (2002). Increased error-prone non homologous DNA end-joining-a proposed mechanism of chromosomal instability in Bloom′s syndrome. Oncogene.

[24] Rassool FV (2003). DNA double strand breaks (DSB) and non-homologous end joining (NHEJ) pathways in human leukemia. Cancer Lett..

[25] Jekimovs C (2014). Chemotherapeutic compounds targeting the DNA double-strand break repair pathways: the good, the bad, and the promising. Front Oncol..

[26] Antonysamy S (2012). Crystal structure of the human PRMT5:MEP50 complex. Proc. Natl Acad. Sci. USA.

[27] Pal S (2003). mSin3A/histone deacetylase 2- and PRMT5-containing Brg1 complex is involved in transcriptional repression of the Myc target gene cad. Mol. Cell Biol..

[28] Sun L (2011). Structural insights into protein arginine symmetric dimethylation by PRMT5. Proc. Natl Acad. Sci. USA.

[29] Ho MC (2013). Structure of the arginine methyltransferase PRMT5-MEP50 reveals a mechanism for substrate specificity. PLoS ONE.

[30] Martin GS (2001). The hunting of the Src. Nat. Rev. Mol. Cell Biol..

[31] Yeatman TJ (2004). A renaissance for SRC. Nat. Rev. Cancer.

[32] Hu D (2015). Interplay between arginine methylation and ubiquitylation regulates KLF4-mediated genome stability and carcinogenesis. Nat. Commun..

[33] Boisvert FM, Rhie A, Richard S, Doherty AJ (2005). The GAR motif of 53BP1 is arginine methylated by PRMT1 and is necessary for 53BP1 DNA binding activity. Cell Cycle.

[34] Liu L (2016). Arginine methylation of SREBP1a via PRMT5 promotes de novo lipogenesis and tumor growth. Cancer Res..

[35] Hu Y (2014). Regulation of 53BP1 protein stability by RNF8 and RNF168 is important for efficient DNA double-strand break repair. PLoS ONE.

[36] Hamard PJ (2018). PRMT5 regulates DNA repair by controlling the alternative splicing of histone-modifying enzymes. Cell Rep..

[37] Clarke TL (2017). PRMT5-dependent methylation of the TIP60 coactivator RUVBL1 Is a key regulator of homologous recombination. Mol. Cell.

[38] Deriano L, Roth DB (2013). Modernizing the nonhomologous end-joining repertoire: alternative and classical NHEJ share the stage. Annu Rev. Genet..

[39] Daley JM, Sung P (2014). 53BP1, BRCA1, and the choice between recombination and end joining at DNA double-strand breaks. Mol. Cell Biol..

[40] Soo Lee N (2016). TRAIP/RNF206 is required for recruitment of RAP80 to sites of DNA damage. Nat. Commun..

[41] Bennardo N, Cheng A, Huang N, Stark JM (2008). Alternative-NHEJ is a mechanistically distinct pathway of mammalian chromosome break repair. PLoS Genet..

[42] Botuyan MV (2006). Structural basis for the methylation state-specific recognition of histone H4-K20 by 53BP1 and Crb2 in DNA repair. Cell.

[43] Pak MG, Lee HW, Roh MS (2015). High nuclear expression of protein arginine methyltransferase-5 is a potentially useful marker to estimate submucosal invasion in endoscopically resected early colorectal carcinoma. Pathol. Int.

[44] Yang H (2016). PRMT5 competitively binds to CDK4 to promote G1-S transition upon glucose induction in hepatocellular carcinoma. Oncotarget.

[45] Deng X (2017). Protein arginine methyltransferase 5 functions as an epigenetic activator of the androgen receptor to promote prostate cancer cell growth. Oncogene.

[46] Betermier M, Bertrand P, Lopez BS (2014). Is non-homologous end-joining really an inherently error-prone process?. PLoS Genet..

[47] Squatrito M, Vanoli F, Schultz N, Jasin M, Holland EC (2012). 53BP1 is a haploinsufficient tumor suppressor and protects cells from radiation response in glioma. Cancer Res.

[48] Iwabuchi K (2006). 53BP1 contributes to survival of cells irradiated with X-ray during G1 without Ku70 or Artemis. Genes Cells.

[49] Adams MM (2005). 53BP1 oligomerization is independent of its methylation by PRMT1. Cell Cycle.

[50] Yu Z, Chen T, Hebert J, Li E, Richard S (2009). A mouse PRMT1 null allele defines an essential role for arginine methylation in genome maintenance and cell proliferation. Mol. Cell Biol..

[51] Huyen Y (2004). Methylated lysine 79 of histone H3 targets 53BP1 to DNA double-strand breaks. Nature.

[52] Pryde F (2005). 53BP1 exchanges slowly at the sites of DNA damage and appears to require RNA for its association with chromatin. J. Cell Sci..

[53] Han X (2014). UbcH7 regulates 53BP1 stability and DSB repair. Proc. Natl Acad. Sci. USA.

[54] Zhang F (2020). Nudix hydrolase NUDT16 regulates 53BP1 protein by reversing 53BP1 ADP-ribosylation. Cancer Res.

[55] Pennington KP (2013). 53BP1 expression in sporadic and inherited ovarian carcinoma: relationship to genetic status and clinical outcomes. Gynecol. Oncol..

[56] Jacot W (2013). BRCA1 promoter hypermethylation, 53BP1 protein expression and PARP-1 activity as biomarkers of DNA repair deficit in breast cancer. BMC Cancer.

[57] Mayca Pozo F (2017). Regulatory cross-talk determines the cellular levels of 53BP1 protein, a critical factor in DNA repair. J. Biol. Chem..

