# Supplementary material for: PRMT5 promotes DNA repair through methylation of 53BP1 and is regulated by Src-mediated phosphorylation
Source: Commun Biol. 2020 Aug 5;3:428. doi: 10.1038/s42003-020-01157-z (PMC7406651; doi:10.1038/s42003-020-01157-z)
Supplement: Supplementary file 1 — Supplementary figure 1 [file 42003_2020_1157_MOESM1_ESM.pdf]

Supplementary Figure 1

a

Motif	Motifgroup	Site	Sequence	Surface Accessibility	Gene Info
Akt Kinase (Akt_Kin)	Basophilic serine/threonine kinase (Baso_ST_kin)	T634	NPTGRSYtIGL****	0.3973	AKT1
ATM Kinase (ATM_Kin)	DNA damage kinase group (DNA_dam_kin)	S446	SFADNELsPECLDGA	0.6296	ATM
PDGFR Kin (PDGFR_Kin)	Tyrosine kinase group (Y_kin)	Y324	DNLESQTyEVFEKDP	0.9989	PDGFRB
Src Kinase (Src_Kin)	Tyrosine kinase group (Y_kin)	Y324	DNLESQTyEVFEKDP	0.9989	SRC

b

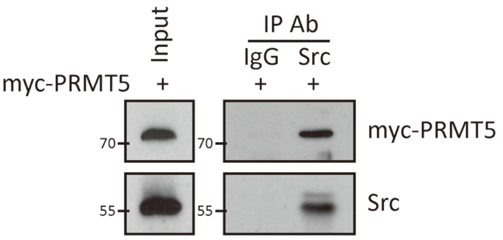

c

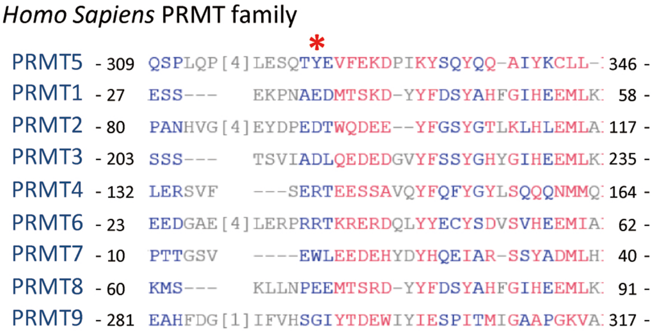

d

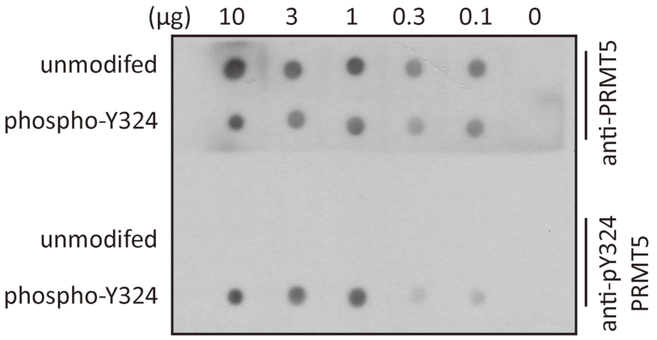

e

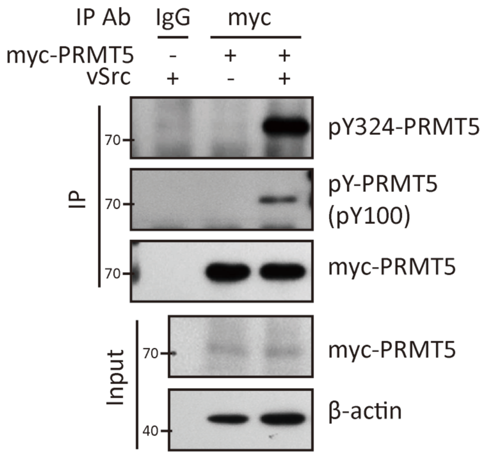

f

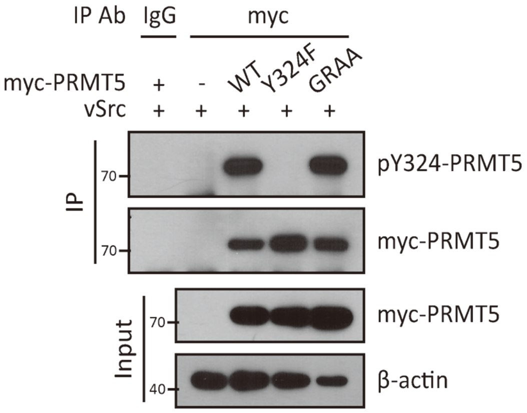

g

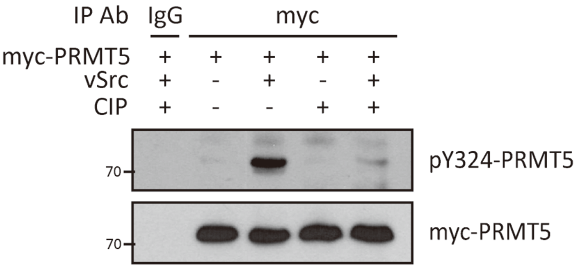

h

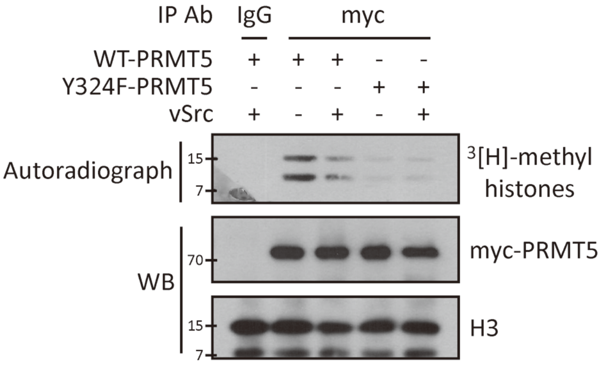

i

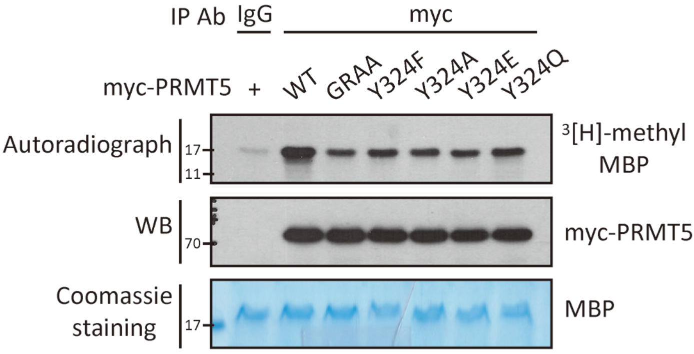

Supplementary Figure 1. Development of phospho-Y324 PRMT5-specific antibody.

(a) Scansite database (https://scansite4.mit.edu/4.0) result using human PRMT5 amino acid query sequence (accession number: O14744). The score of surface accessibility indicates how close the predicted residues are to the protein surface. **(b)** Interaction between myc-PRMT5 and Src kinase. U2OS cells were transfected with myc-PRMT5 and then subjected to co-IP using Src kinase antibody. **(c)** Protein domain alignment of the members of human PRMT family using COBALT (Constraint-based Multiple Alignment Tool, https://www.ncbi.nlm.nih.gov/tools/cobalt) database. The red color indicates highly conserved columns and blue indicates less conserved ones. Red asterisk indicates Y324 residue of PRMT5. **(d)** Dot blot assay using phospho-Y324 PRMT5-specific antibody. Serial amounts of the unmodified and Y324-phosphorylated PRMT5 peptide were spotted on PVDF membrane and detected by pY324-PRMT5 antibody. **(e** and **f)** Validation of pY324-PRMT5 antibody. HEK293T cells were transfected with myc-PRMT5 alone or in combination with vSrc (e), or co-transfected with myc-PRMT5 (WT, Y324F, or GRAA) and vSrc (f) for 1 day. Myc-PRMT5 was immunoprecipitated and immunoblotted using pY324-PRMT5 antibody. **(g)** CIP assay for vSrc-mediated PRMT Y324 phosphorylation. HEK293T cells were co-transfected with myc-PRMT5 and vSrc plasmids, and then myc-PRMT5 protein was immunoprecipitated. The myc-PRMT5 was incubated with calf intestinal phosphatase (CIP). **(h)** *In vitro* PRMT5 methyltransferase assay using recombinant histone proteins. Immunoprecipitated myc-PRMT5 from HEK293T cells was incubated with 1 μg recombinant histone proteins (mixture of H2A, H2B, H3, and H4) and 1 μCi ^{3}[H]-AdoMet at 37 ºC for 1 h. Methylated samples were analyzed by SDS–PAGE and autoradiography as described. **(i)** Comparison of methyltransferase activity between Y324 substitution mutants. WT, Y324F, Y324A, Y324E, Y324Q, and GRAA myc-PRMT5 were used to perform *in vitro* methyltransferase assay. The immunoblots in (b, d-i), autoradiographs in (h, i) and CBB in (i) are representative of three independent experiments with similar results, respectively.

Supplementary Figure 2

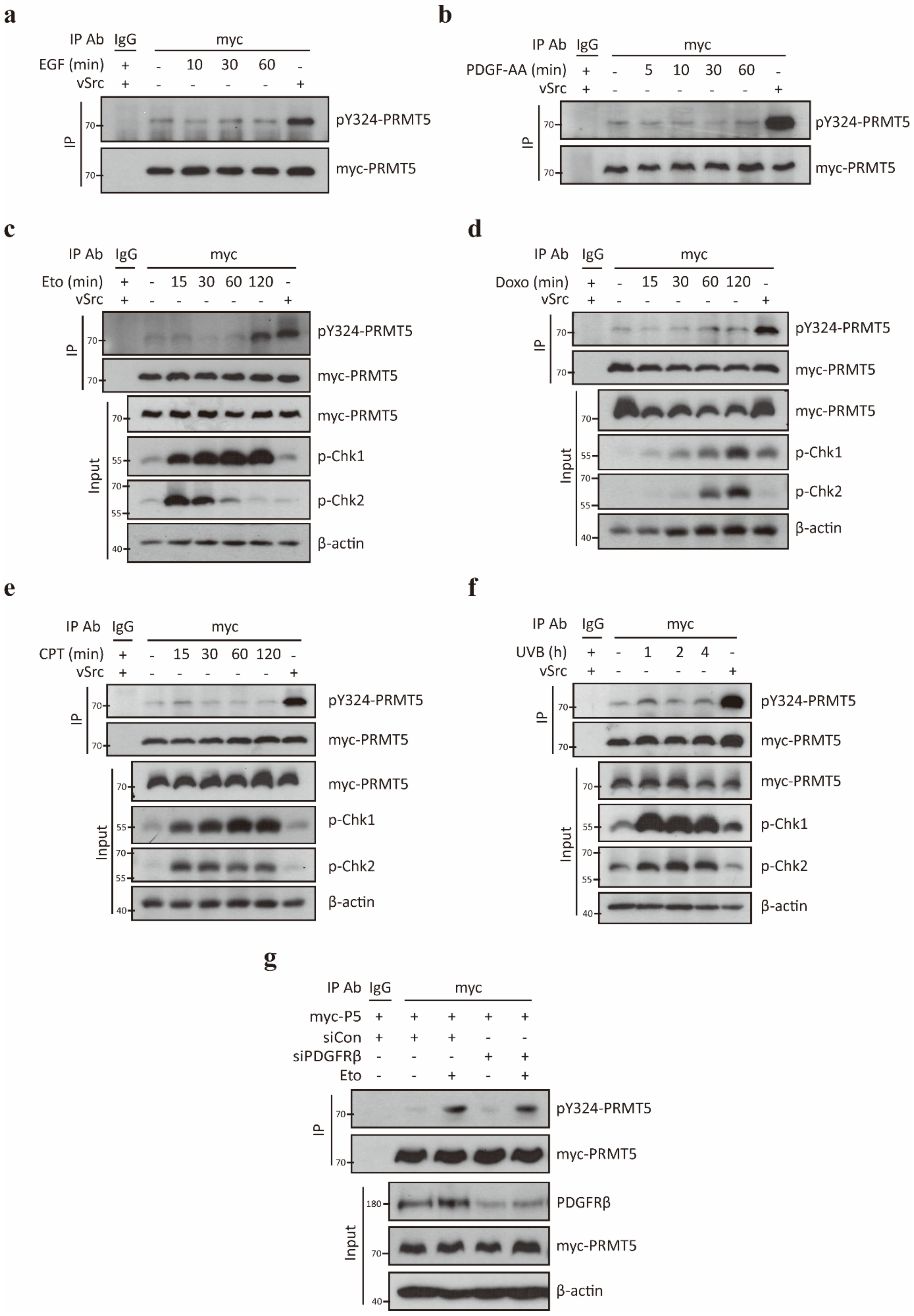

Supplementary Figure 2. Y324 phosphorylation of PRMT5 occurs under DNA damage stress.

(a, b) The Y324 phosphorylation of PRMT5 is not induced by either EGF or PDGF-AA. A549 cells were transfected with myc-PRMT5 and then treated with 10 ng/ml EGF (a) and NIH3T3 cells were transfected with myc-PRMT5 and treated with 20 ng/ml PDGF-AA (b) for the indicated times. Immunoprecipitated myc-PRMT5 was detected by immunoblotting using pY324-PRMT5 antibody. **(c - f)** U2OS cells were transfected with myc-PRMT5 and then treated with 50 μM etoposide (c), 1 μM doxorubicin (d), or 10 μM camptothecin (CPT) (e) for indicated times. (f) After stimulation with 20 J/cm^2 UVB, myc-PRMT5 expressing U2OS cells were incubated for indicated times. Myc-PRMT5 was immunoprecipitated, followed by immunoblotting with pY324-PRMT5 antibody. **(g)** PDGFRβ is not responsible for etoposide-induced Y324 phosphorylation of PRMT5. U2OS cells were co-transfected with myc-PRMT5 plasmid and human PDGFRβ-targeting siRNA duplex for 3 days, and then treated with 50 μM etoposide for 2 h. All immunoblots in Supplementary Fig. 2 are representative of three independent experiments with similar results, respectively.

Supplementary Figure 3

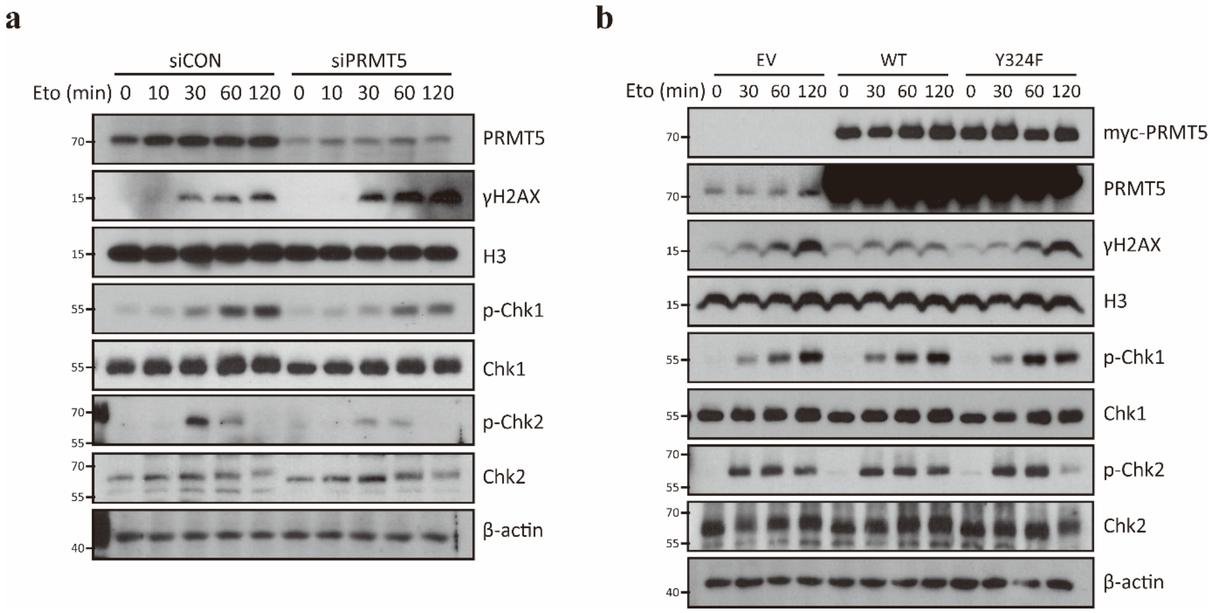

Supplementary Figure 3. Regulation of DNA damage signaling by PRMT5

(a, b) Role of PRMT5 in DNA damage signaling. U2OS cells were transfected with PRMT5-targeting siRNA for 3 days (a) or myc-PRMT5 (WT or Y324F, b), followed by treatment of 10 μM etoposide for indicated times. Cell lysates were subjected to immunoblotting analysis for the indicated antibodies. All immunoblots in Supplementary Fig. 3 are representative of three independent experiments with similar results, respectively.

Supplementary Figure 4

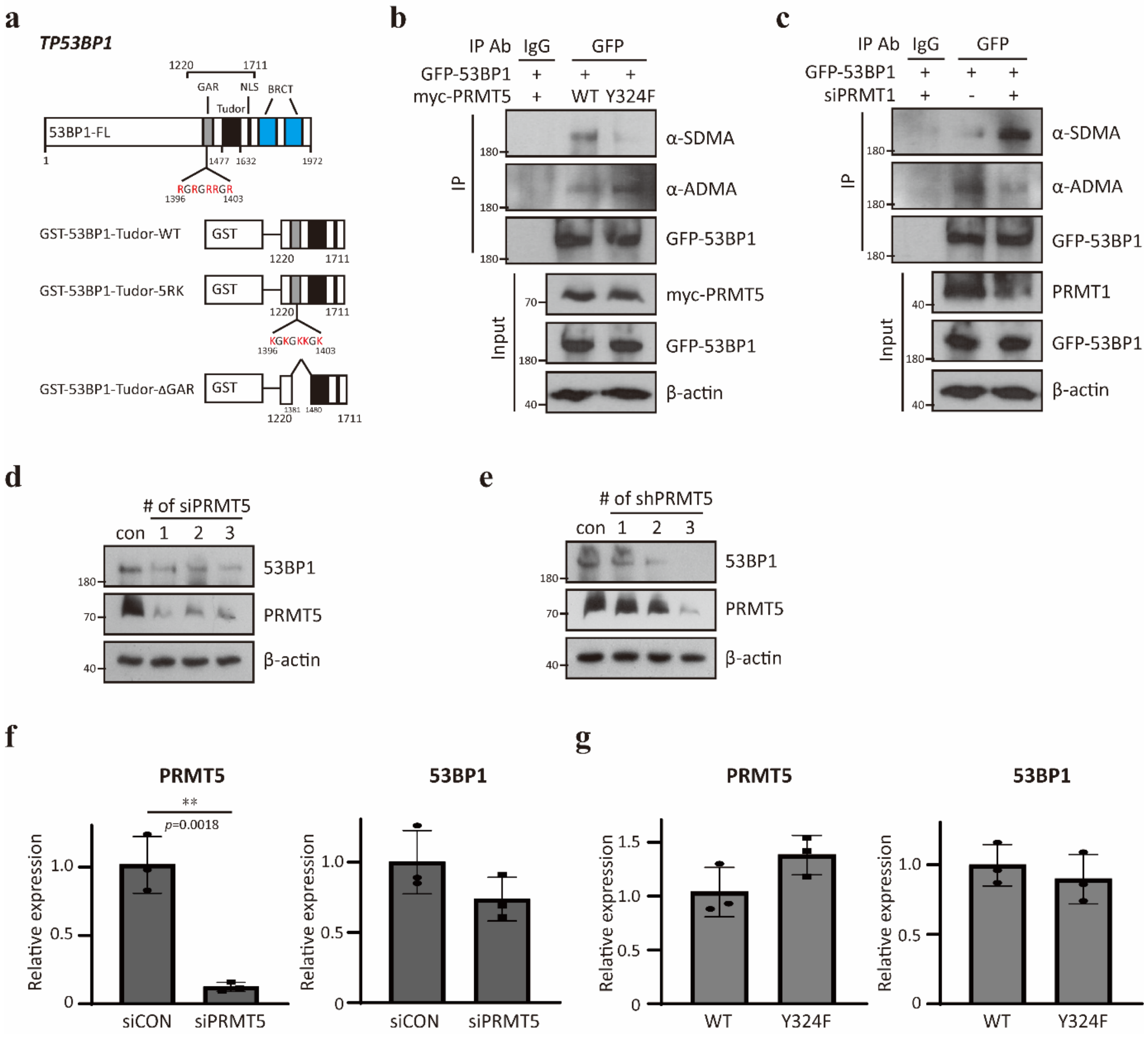

Supplementary Figure 4. Regulation of 53BP1 expression level by PRMT5

(a) Diagram of the domain organization of full length TP53BP1 and GST-53BP1-Tudor (WT, 5RK and ΔGAR) structure used *in vitro* methylation assay in Fig. 4a. GAR (Glycine-Arginine rich motif), Tudor (Tudor domain), NLS (nuclear localization signal), and BRCT (BRCA1 C-terminus domain). **(b, c)** Competition between arginine dimethylation of GAR motif in 53BP1. U2OS cells were transfected with WT or Y324F myc-PRMT5 (b) or PRMT1-targeting siRNA (c) for 2 days. GFP-53BP1 was immunoprecipitated by GFP antibody and then immunoblotted using anti-ADMA or anti-SDMA antibody. **(d, e)** U2OS cells were transfected with three different siPRMT5s (d) or shPRMT5s (e) for 3 days. Whole lysates were analyzed by immunoblot assays. The immunoblots in (b - e) are representative of three independent experiments with similar results, respectively. **(f, g)** Expression levels of 53BP1 mRNA. PRMT5-depleted (f) or myc-PRMT5 overexpressed (g) U2OS cells were subjected to quantitative PCR assay. Error bars represent standard deviation (n=3, $^{**}p < 0.01$).

Supplementary Figure 5

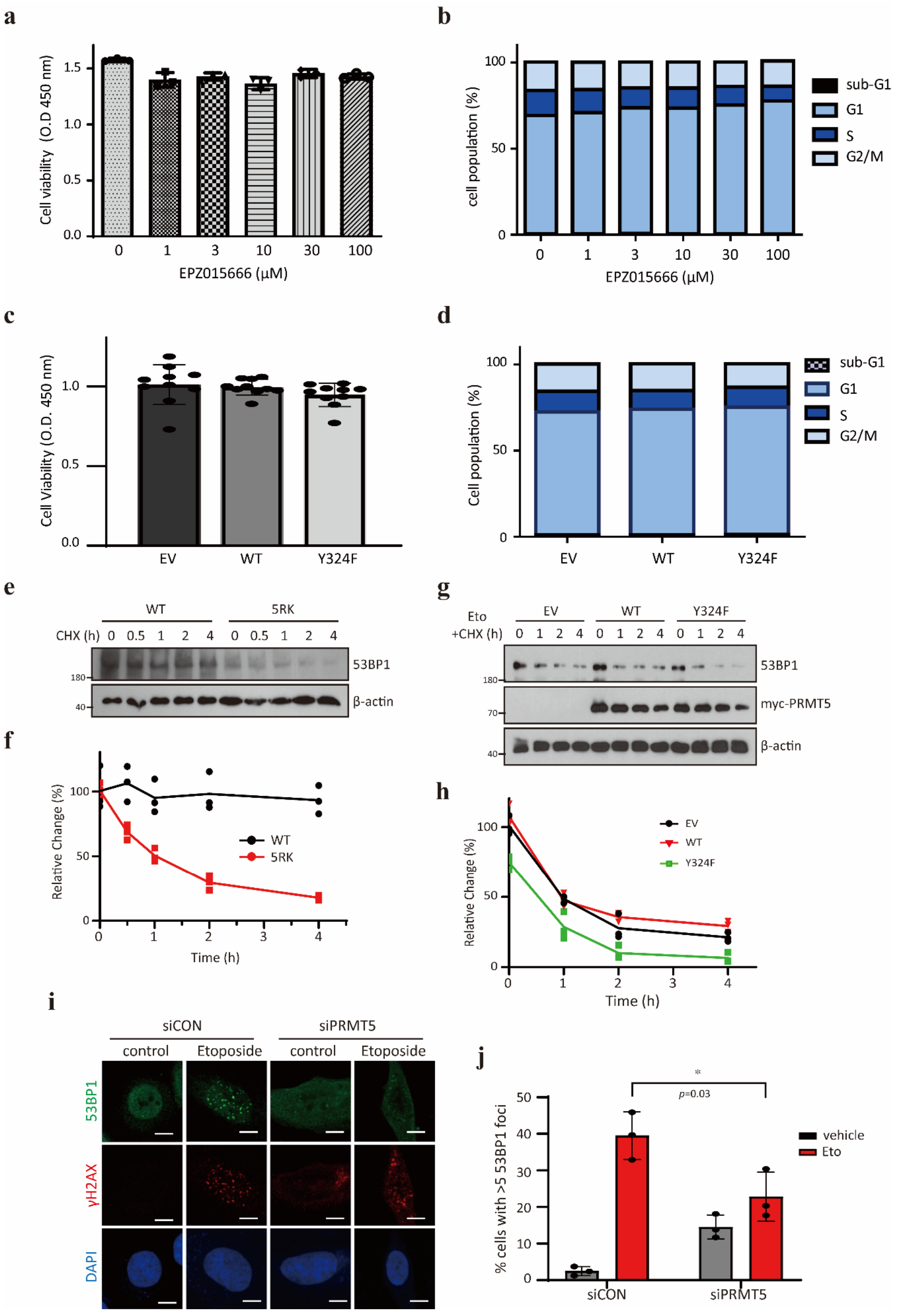

Supplementary Figure 5. PRMT5 regulates 53BP1 protein stability

(a - d) Analysis of cell viability and cell cycle. U2OS cells were treated with EPZ01566 (a, b) or transfected with WT or Y324F myc-PRMT5 (c, d) for 2 days. Cells were subjected to MTS cell viability assay (a, c) or PI staining for cell cycle analysis (b, d). Error bars represent standard deviation (n=10). **(e - h)** Analysis of 53BP1 protein stability. U2OS cells were transfected with WT or 5RK GFP-53BP1 (e, f) for 1 day and then treated with 50 μg/ml cycloheximide (CHX) for the indicated times. (g, h) Myc-PRMT5 transfected U2OS cells were co-treated with 50 μg/ml CHX and 10 μM etoposide for the indicated times. Immunoblotting data in (e, g) are representative of three independent experiments. The 53BP1 band intensities were quantitated using image processing software (f, h, n=3). **(i - j)** Analysis of nuclear 53BP1 foci formation. U2OS cells depleted endogenous PRMT5 were assessed by co-immunostaining for 53BP1 and γH2AX, followed by DAPI staining. (i) Nuclear 53BP1 foci were analyzed by confocal microscopy. Scale Bar: 5μm. (j) The percentage of cells with ≥ 5 53BP1 foci in (i) is shown. Error bars represent standard deviation (n=3, $*p < 0.05$).

Supplementary Figure 6

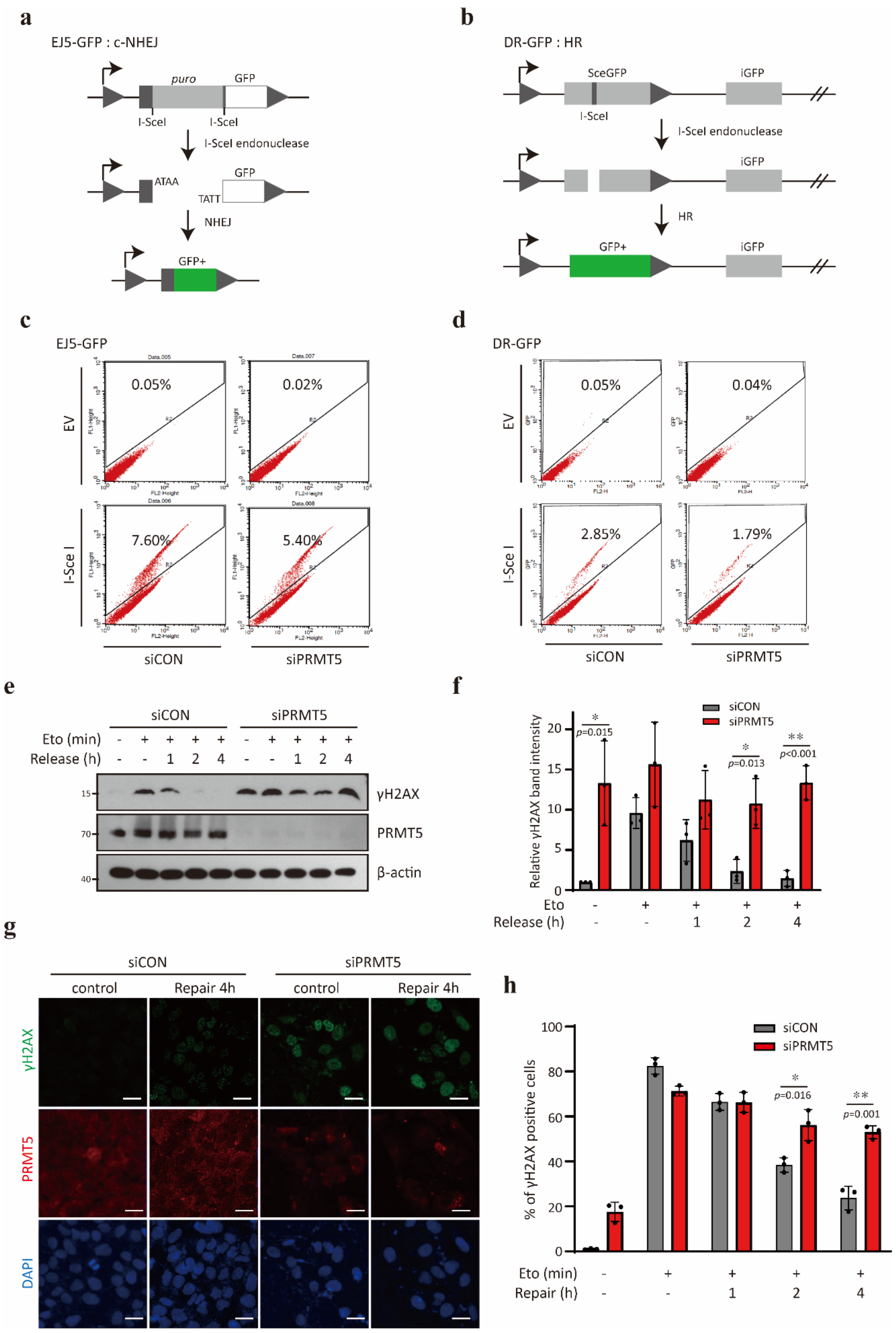

Supplementary Figure 6. Involvement of PRMT5 in DNA repair process

(a, b) Schematic of the EJ5-GFP and DR-GFP reporter assays. (a) EJ5-GFP reporter system used to monitor total NHEJ process. GFP gene is separated from its promoter by a puromycin resistance gene (*puro*) flanked by two I-SceI sites. Transient expression of I-SceI endonuclease causes the excision of the *puro* and induces the repair of the DSB by NHEJ thus creating a functional GFP gene. (b) DR-GFP reporter system used to monitor HR process. Modified GFP gene (SceGFP) contains an I-SceI site inside frame. Expression of I-SceI causes the DBS at internal SceGFP gene and a non-functional GFP fragment gene (iGFP) is used to repair the DSB by HR, generating a functional GFP gene. **(c, d)** U2OS stable cell lines expressing EJ5-GFP (c) or DR-GFP (d) reporter constructs were co-transfected with PRMT5 siRNA and I-SceI plasmid. After 3 days, the efficiency of NHEJ (c) or HR (d) was determined by counting the number of GFP-positive cells using flow cytometry. The mean of percentage of GFP-positive cells from three indicated experiments is indicated. **(e - h)** U2OS cells transfected with PRMT5-targeting siRNA were treated with 10 μM etoposide for 2 h, followed by incubation with fresh media for indicated times. Cells were subjected to immunoblotting (e, f) or immunostaining (g, h) analysis. (e) Representative western bands from three independent experiments are shown. (f) The γH2AX band intensities in (e) were quantitated using image processing software (n=3). (g) Representative pictures are shown. Scale Bar: 20μm. (h) Quantification of cells showing high fluorescence of γH2AX in (g) was done using image processing software (n=3). *: $p < 0.05$ and **: $p < 0.01$.

Supplementary Figure 7

a

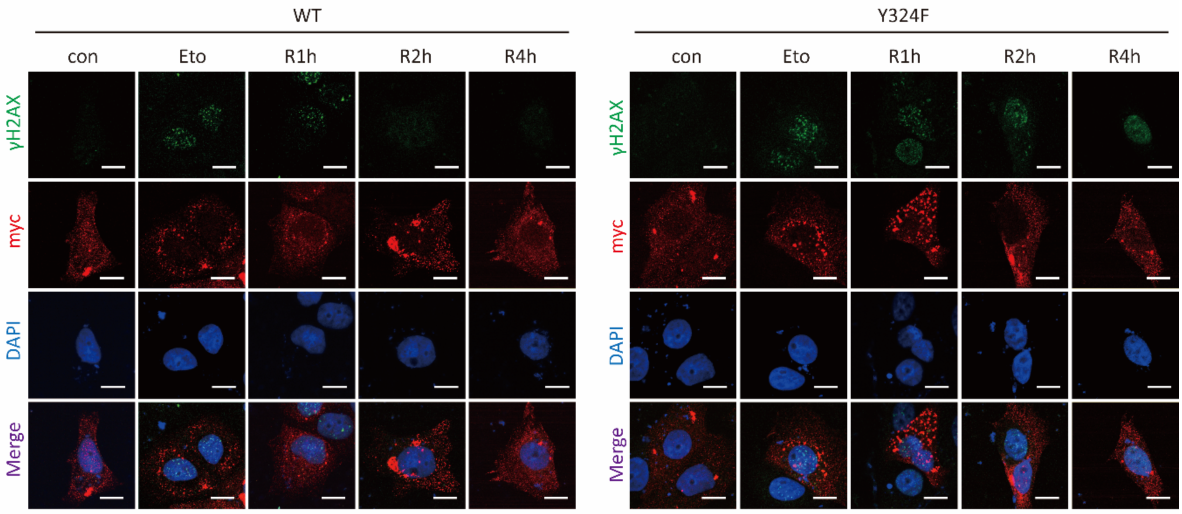

b

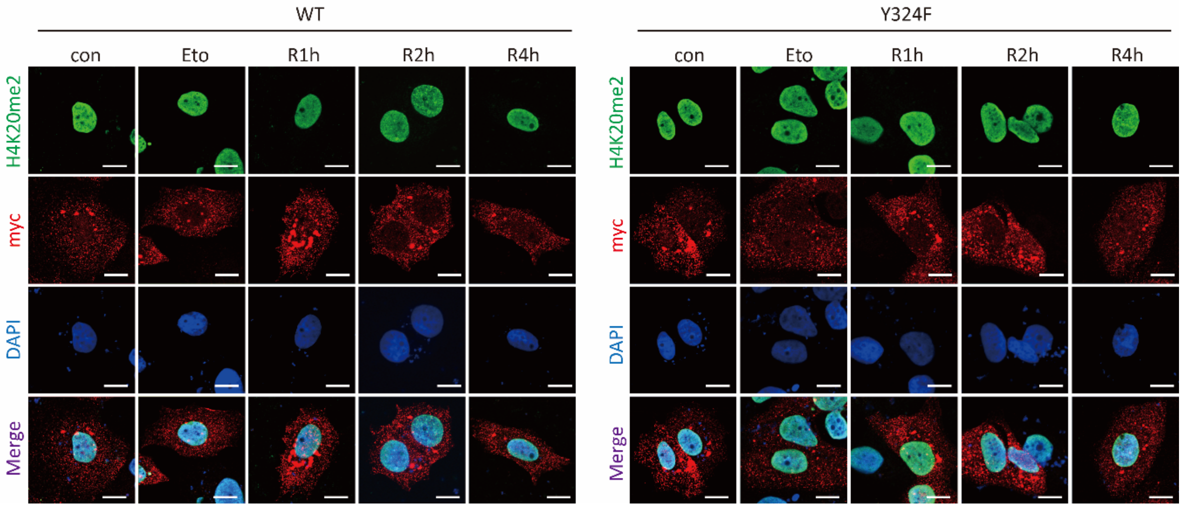

Supplementary Figure 7. Y324F-PRMT5 attenuates the recovery of γH2AX after DNA damage. **(a, b)** WT- or Y324F myc-PRMT5 overexpressing U2OS cells were treated with 10 μM etoposide for 2 h, followed by incubation with fresh media for indicated times. Cells were fixed and then stained with γH2AX (a) or H4K20me2 (b) antibody. Scale Bar: 5 μm.

Supplementary Figure 8

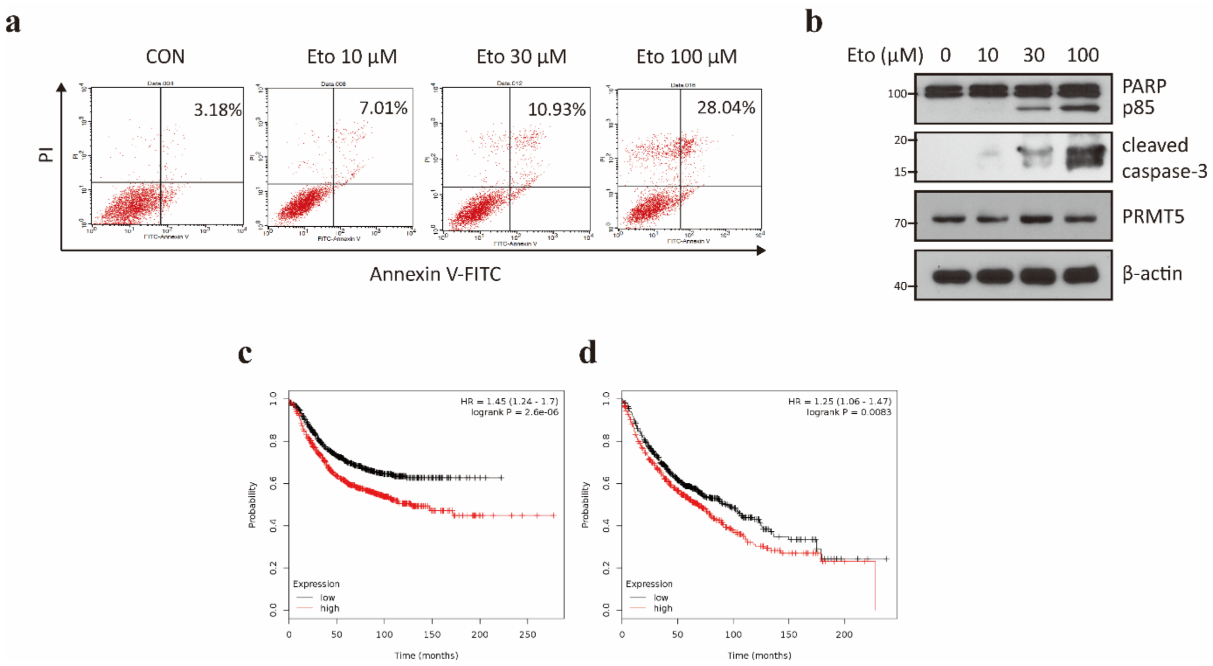

Supplementary Figure 8. PRMT5 levels are linked to survival probability in cancer patients.

(a, b) Etoposide-induced apoptosis with concentration-dependent manner. U2OS cells were treated with etoposide at the indicated concentrations for 24 h and then apoptosis was analyzed by Annexin V/PI staining (a), or PARP and caspase-3 cleavage (b). **(c, d)** A Kaplan–Meier survival probability plot for high (red) versus low (black) PRMT5 gene expression/mRNA level for breast cancer (c) or lung cancer (d) is shown. High PRMT5 expression results in a ~1.4-fold reduction in survival (hazard ratio), with very high significance. Survival data obtained from http://www.kmplot.com.

Fig 1b

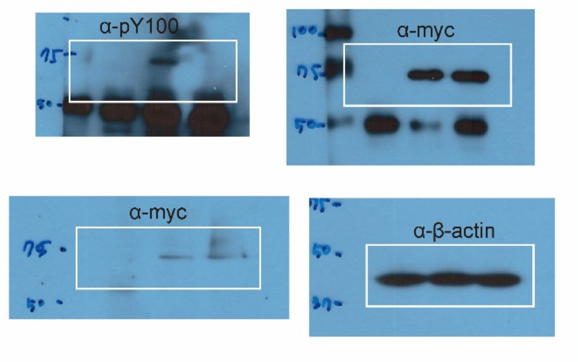

Fig 1c

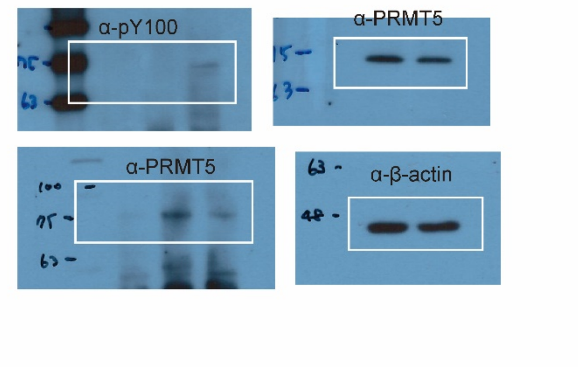

Fig 1d

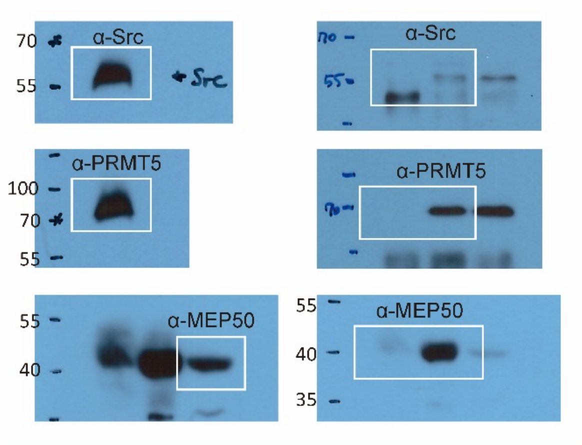

Fig 1e

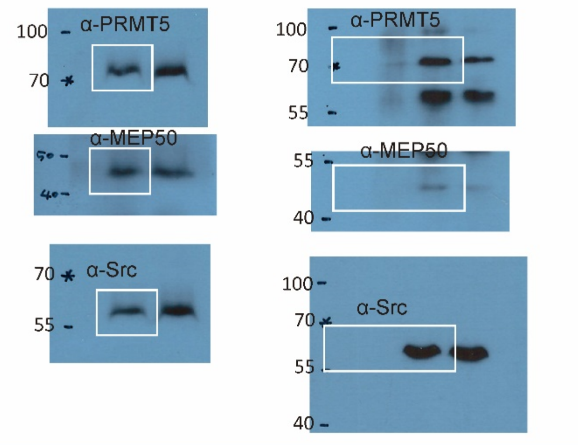

Fig 1f

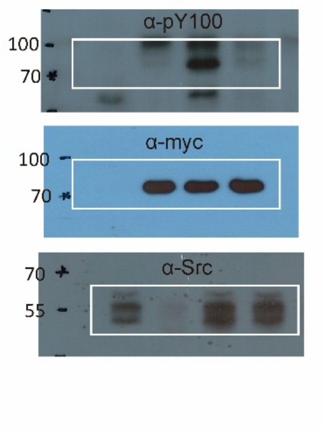

Fig 1g

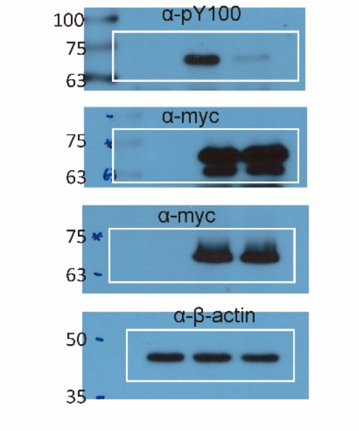

Fig 1i

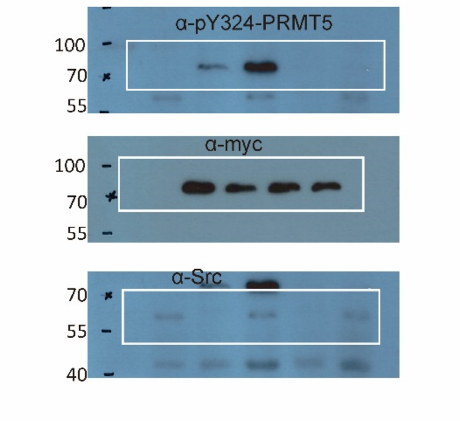

Fig 1j

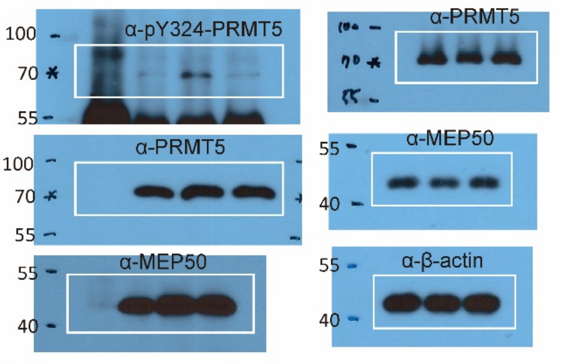

Fig 1k

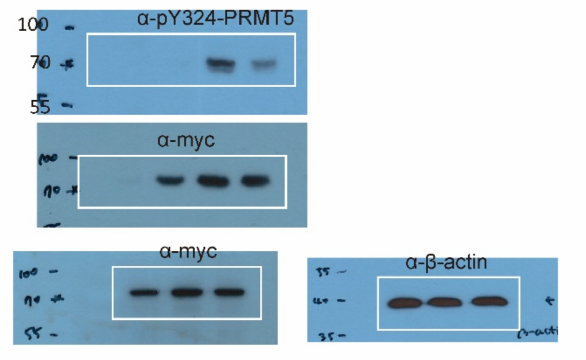

Fig 2b

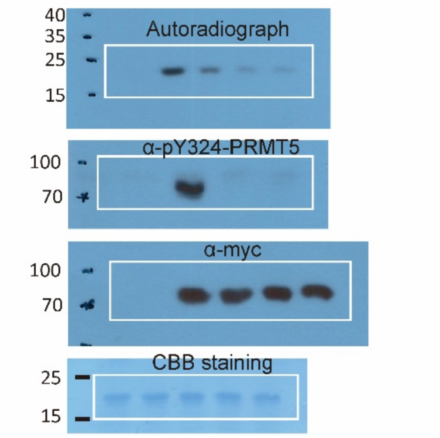

Fig 2c

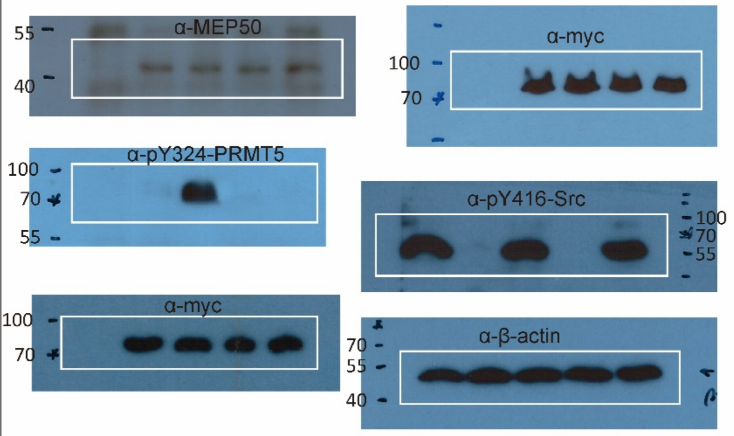

Fig 2d

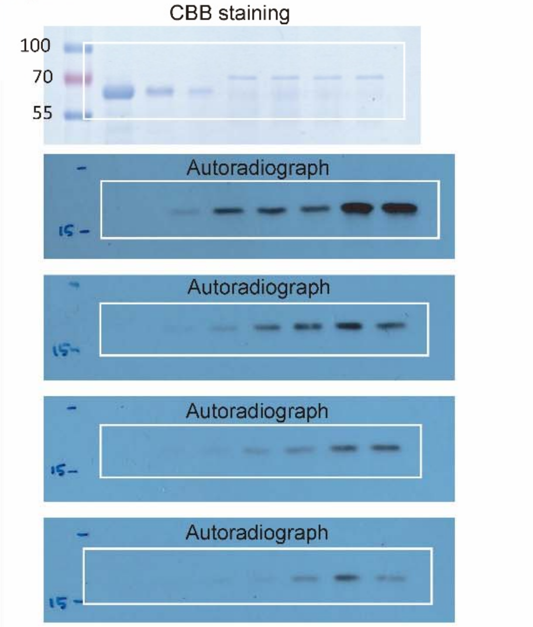

Fig 2g

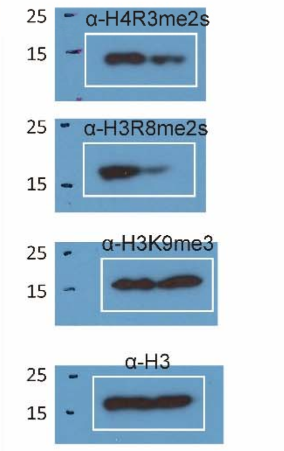

Fig 3a

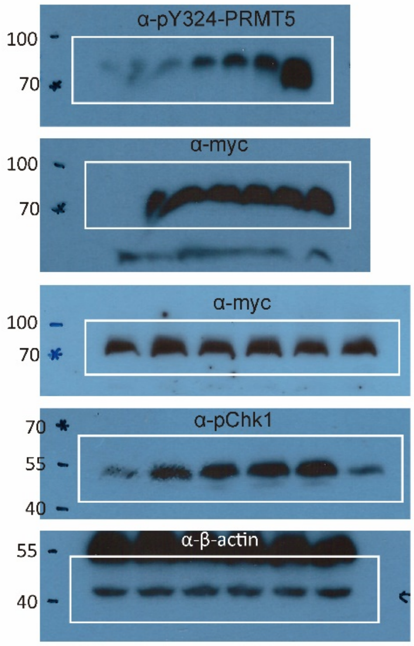

Fig 3b

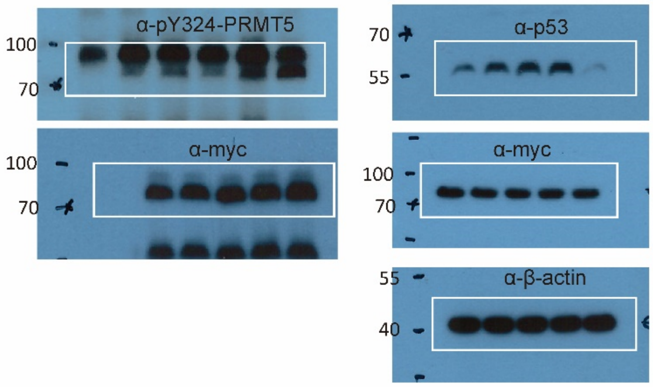

Fig 3c

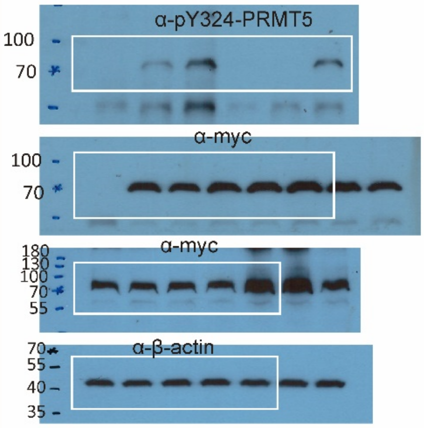

Fig 3d

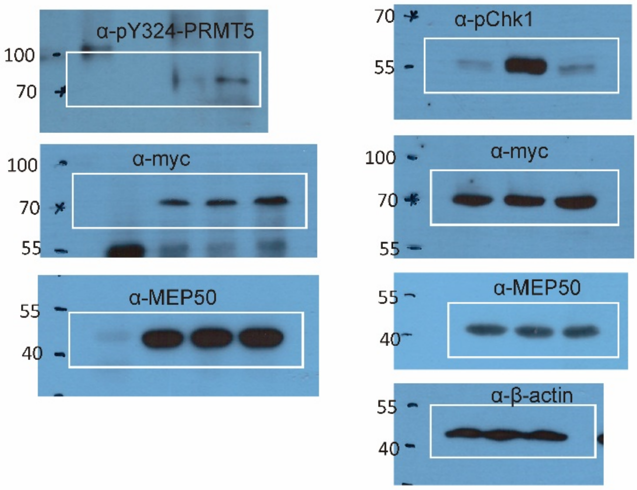

Fig 3d

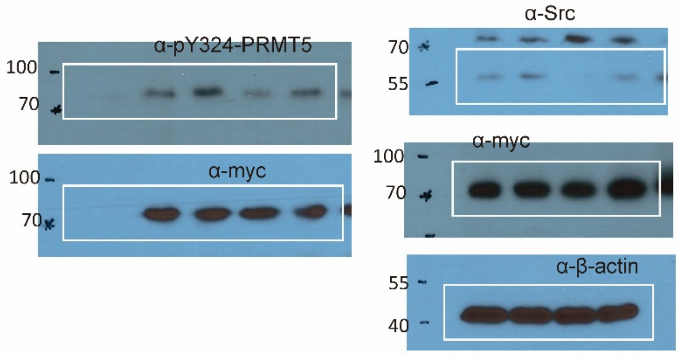

Fig 3e

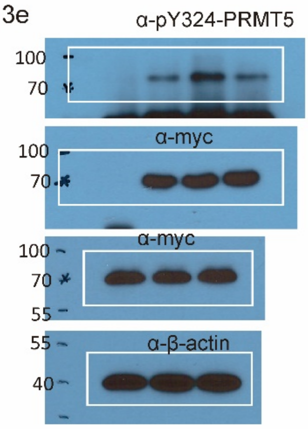

Fig 3g

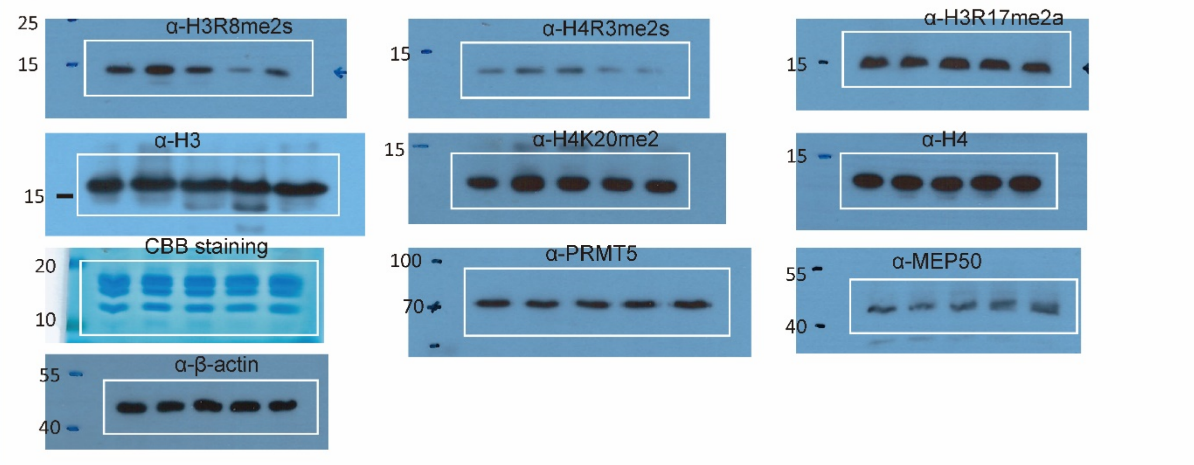

Fig 4a

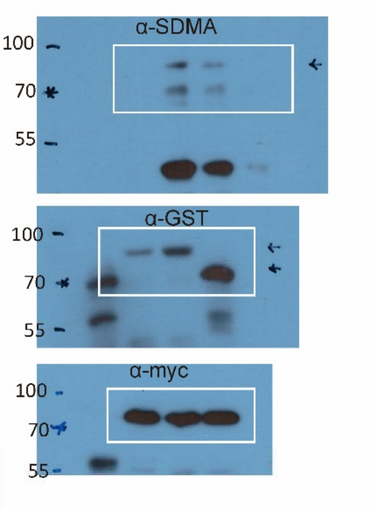

Fig 4b

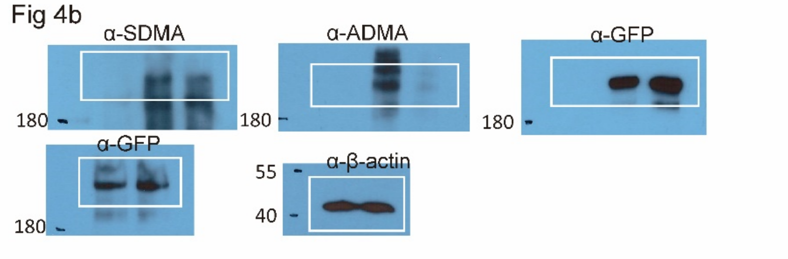

Fig 4c

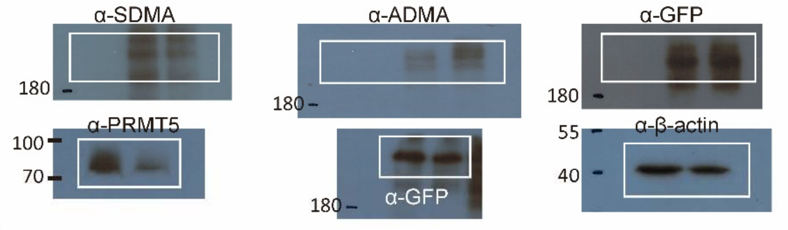

Fig 4d

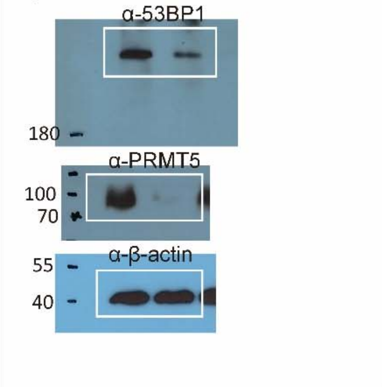

Fig 4f

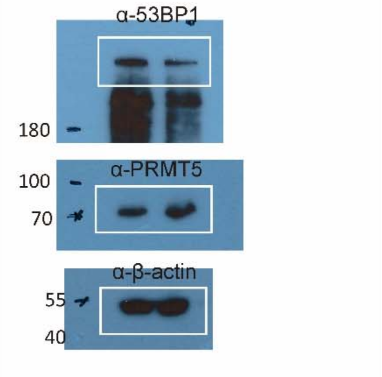

Fig 4h

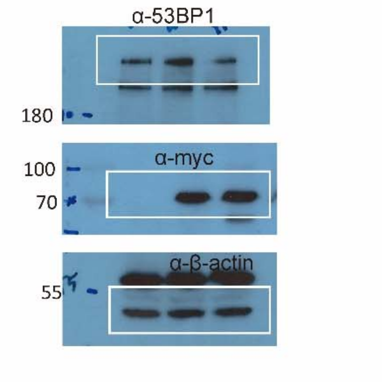

Fig 4j

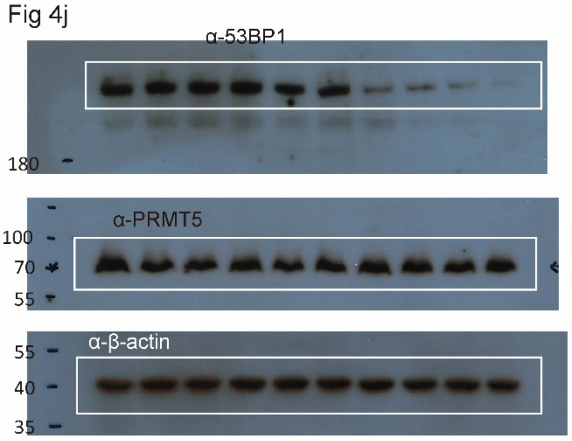

Fig 4l

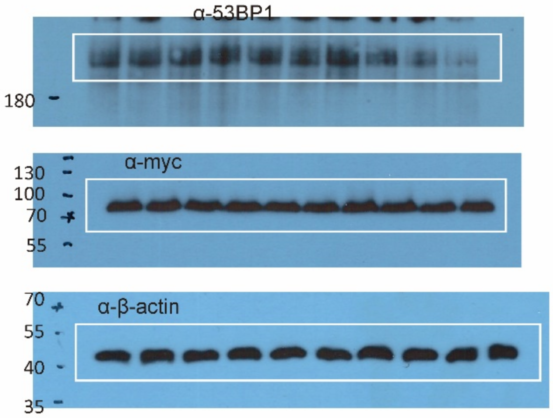

Fig 5c

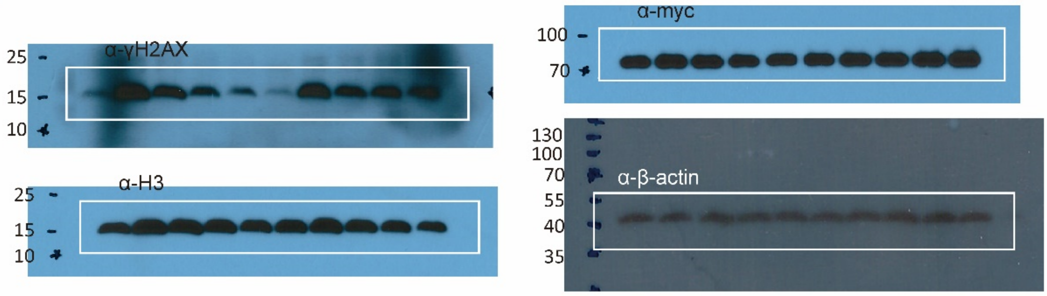

Fig 5g

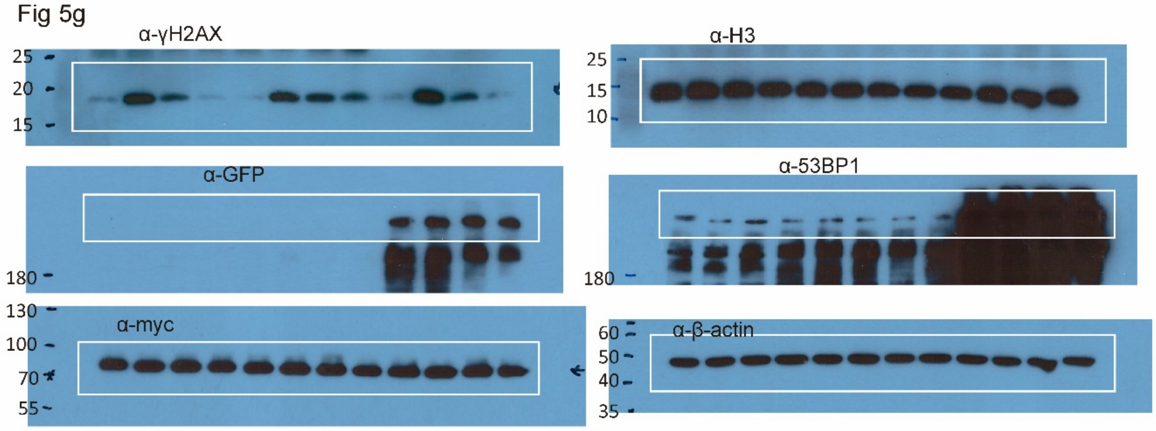

Fig S1b

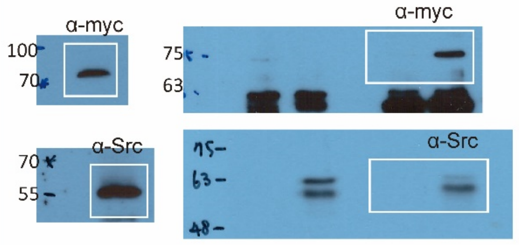

Fig S1d

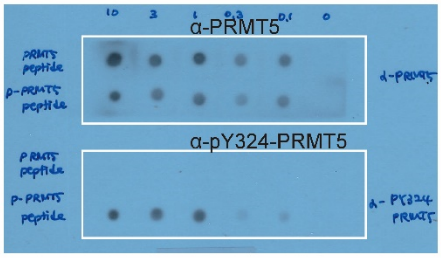

Fig S1e

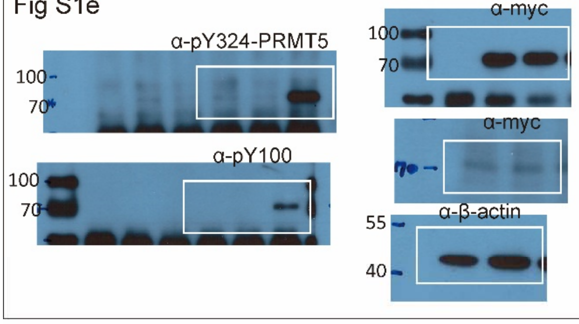

Fig S1f

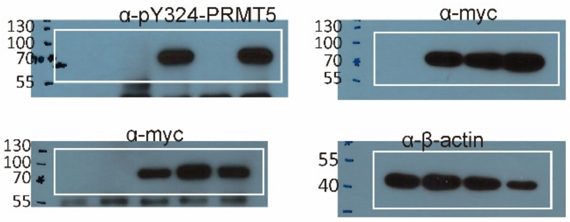

Fig S1g

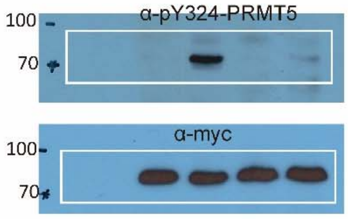

Fig S1h

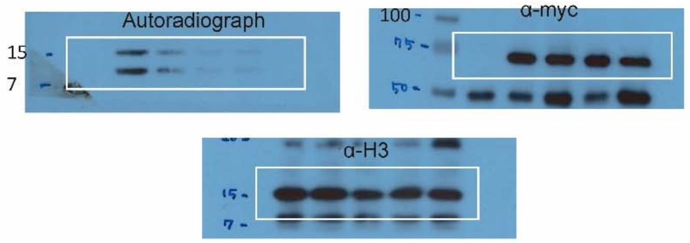

Fig S1i

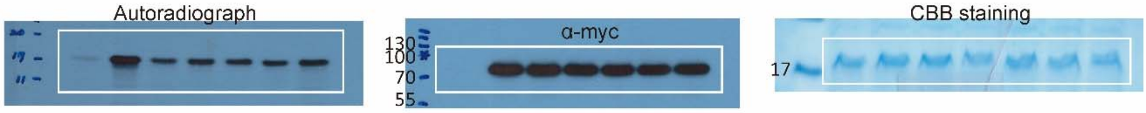

Fig S2a

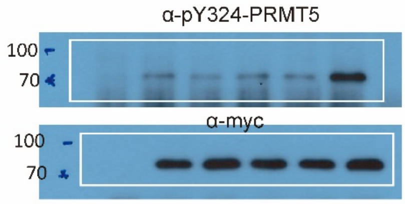

Fig S2b

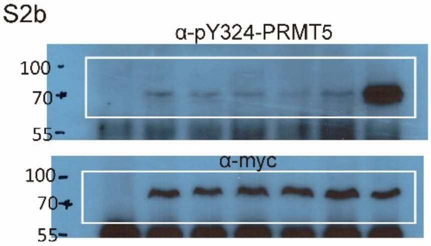

Fig S2c

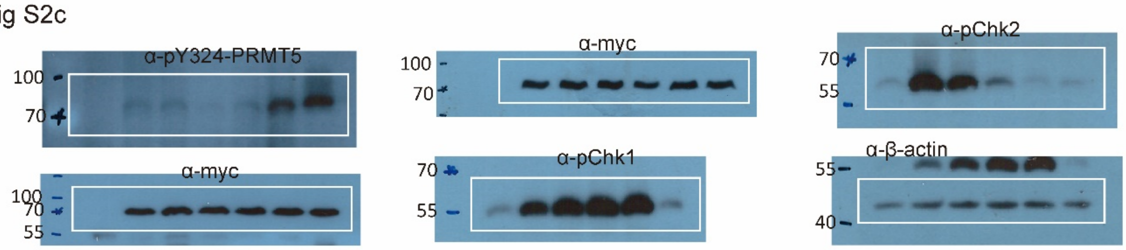

Fig S2d

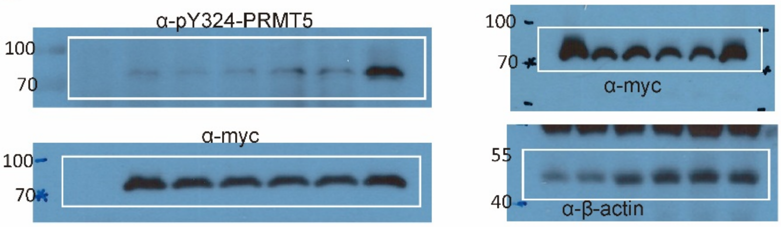

Fig S2e

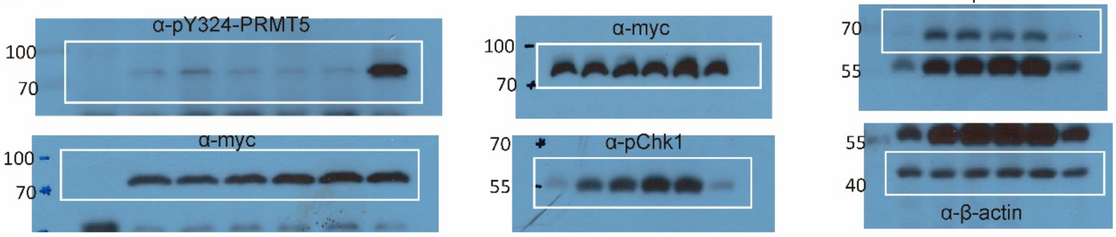

Fig S2f

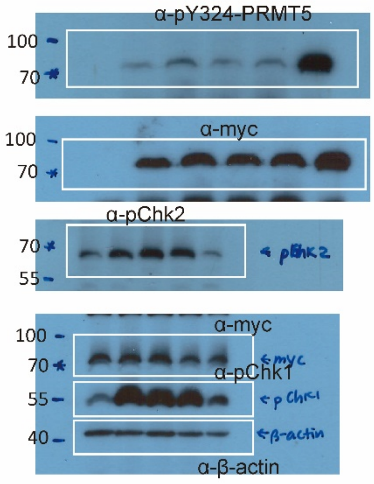

Fig S2g

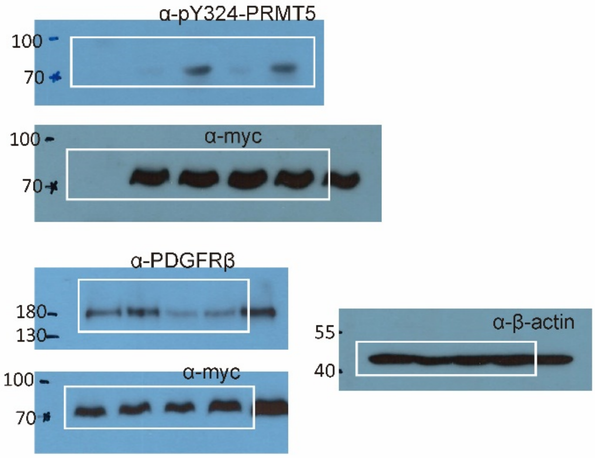

Fig S3a

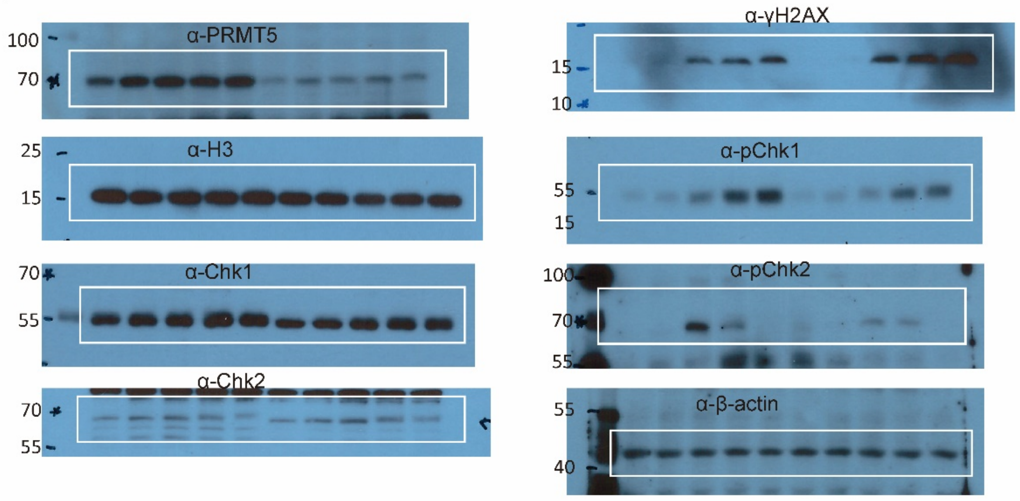

Fig S3b

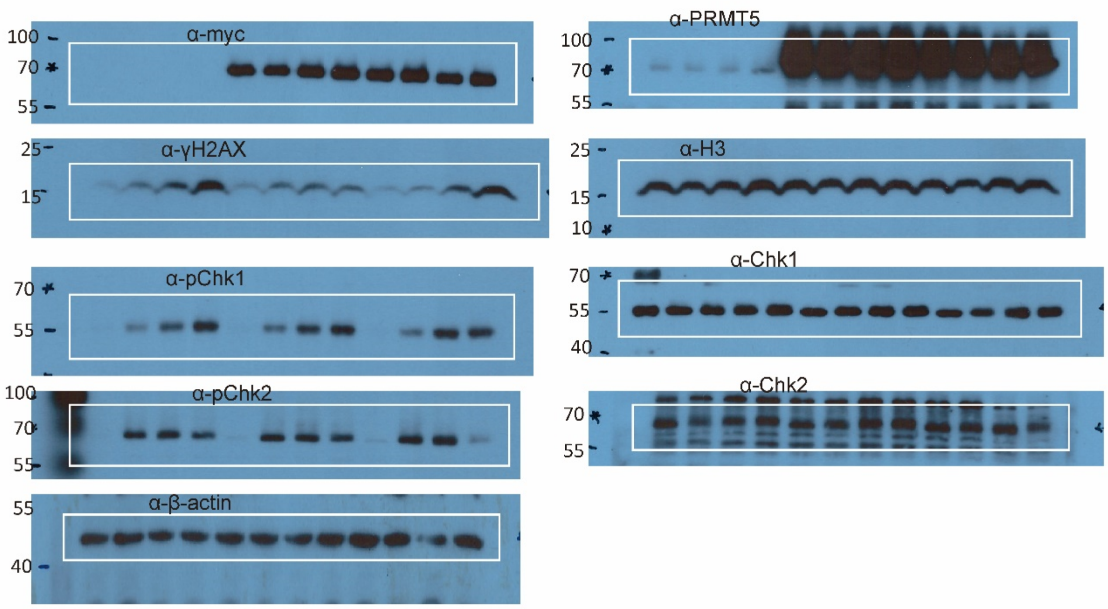

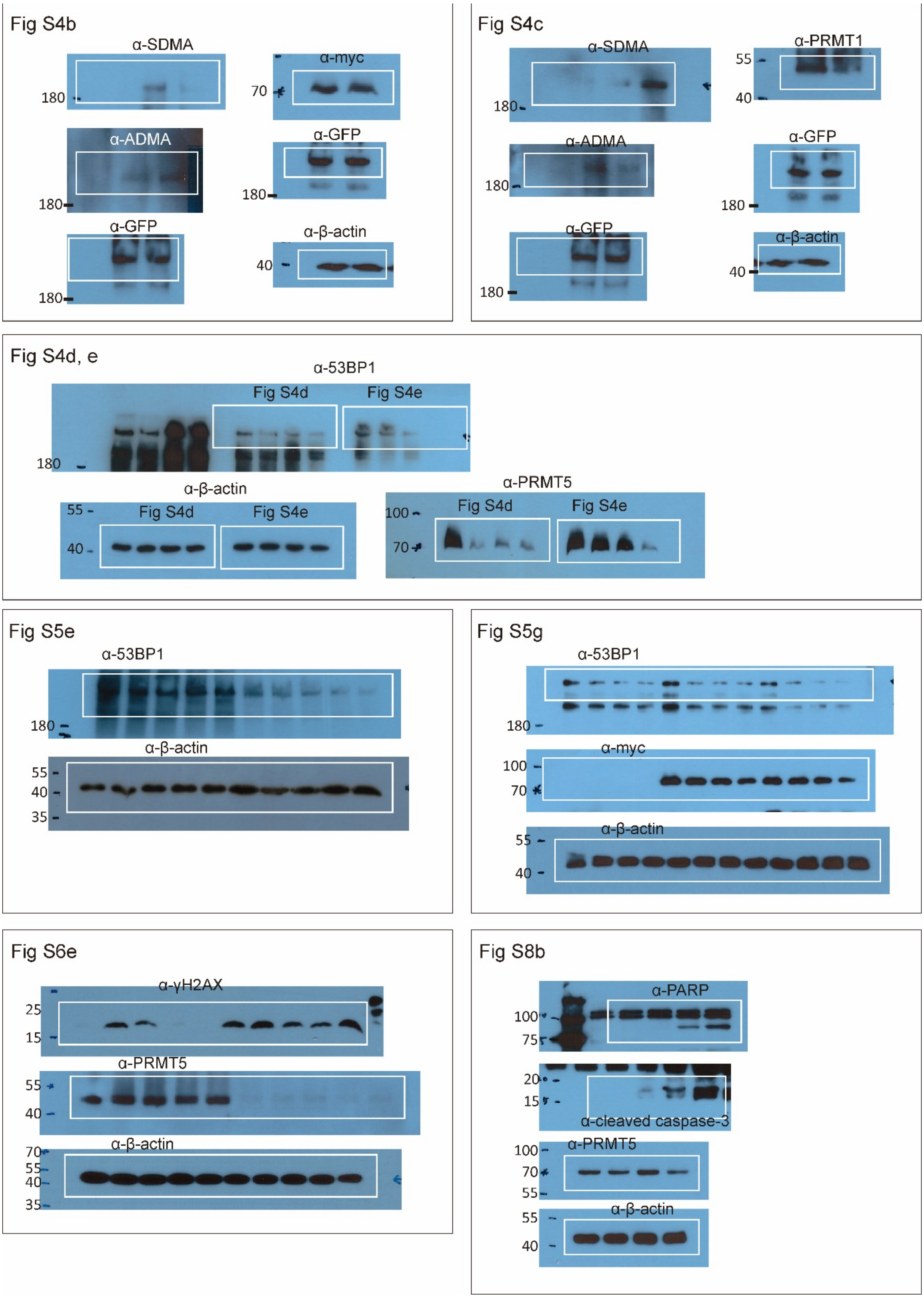
Fig S4b
α-SDMA
180
α-myc
70
α-ADMA
180
α-GFP
180
α-GFP
180
α-β-actin
40
Fig S4c
α-SDMA
180
α-PRMT1
55
40
α-ADMA
180
α-GFP
180
α-GFP
180
α-β-actin
40
Fig S4d, e
α-53BP1
Fig S4d
Fig S4e
180
α-β-actin
55
40
Fig S4d
Fig S4e
α-PRMT5
100
70
Fig S4d
Fig S4e
Fig S5e
α-53BP1
180
α-β-actin
55
40
35
Fig S5g
α-53BP1
180
α-myc
100
70
α-β-actin
55
40
Fig S6e
α-γH2AX
25
15
α-PRMT5
55
40
α-β-actin
70
55
40
35
Fig S8b
α-PARP
100
75
20
15
α-cleaved caspase-3
α-PRMT5
100
70
55
α-β-actin
55
40
