# Supplementary material for: PRMT5 promotes DNA repair through methylation of 53BP1 and is regulated by Src-mediated phosphorylation
Source: Commun Biol. 2020 Aug 5;3:428. doi: 10.1038/s42003-020-01157-z (PMC7406651; doi:10.1038/s42003-020-01157-z)
Supplement: Supplementary file 2 — Supplementary Data 1 [file 42003_2020_1157_MOESM2_ESM.pdf]

## Description of Additional Supplementary Files

**File Name**: **Supplementary Data 1**
**Description**: source data file
