# Supplementary material for: PRMT5 promotes DNA repair through methylation of 53BP1 and is regulated by Src-mediated phosphorylation
Source: Commun Biol. 2020 Aug 5;3:428. doi: 10.1038/s42003-020-01157-z (PMC7406651; doi:10.1038/s42003-020-01157-z)
Supplement: Supplementary file 4 — Reporting Summary [file 42003_2020_1157_MOESM4_ESM.pdf]

nature research

Corresponding author(s): Yong Kee Kim
Last updated by author(s): Jul 3, 2020

Reporting Summary

Nature Research wishes to improve the reproducibility of the work that we publish. This form provides structure for consistency and transparency in reporting. For further information on Nature Research policies, see our Editorial Policies and the Editorial Policy Checklist.

Statistics

For all statistical analyses, confirm that the following items are present in the figure legend, table legend, main text, or Methods section.

n/a	Confirmed	
☐	☒	The exact sample size (*n*) for each experimental group/condition, given as a discrete number and unit of measurement
☐	☒	A statement on whether measurements were taken from distinct samples or whether the same sample was measured repeatedly
☐	☒	The statistical test(s) used AND whether they are one- or two-sided<br>*Only common tests should be described solely by name; describe more complex techniques in the Methods section.*
☐	☒	A description of all covariates tested
☐	☒	A description of any assumptions or corrections, such as tests of normality and adjustment for multiple comparisons
☐	☒	A full description of the statistical parameters including central tendency (e.g. means) or other basic estimates (e.g. regression coefficient) AND variation (e.g. standard deviation) or associated estimates of uncertainty (e.g. confidence intervals)
☐	☒	For null hypothesis testing, the test statistic (e.g. *F*, *t*, *r*) with confidence intervals, effect sizes, degrees of freedom and *P* value noted<br>*Give P values as exact values whenever suitable.*
☒	☐	For Bayesian analysis, information on the choice of priors and Markov chain Monte Carlo settings
☒	☐	For hierarchical and complex designs, identification of the appropriate level for tests and full reporting of outcomes
☒	☐	Estimates of effect sizes (e.g. Cohen's *d*, Pearson's *r*), indicating how they were calculated

Our web collection on statistics for biologists contains articles on many of the points above.

Software and code

Policy information about availability of computer code

Data collection	No unpublished software was used.
Data analysis	All data analyzed using GraphPad Prism (version 8)

For manuscripts utilizing custom algorithms or software that are central to the research but not yet described in published literature, software must be made available to editors and reviewers. We strongly encourage code deposition in a community repository (e.g. GitHub). See the Nature Research guidelines for submitting code & software for further information.

Data

Policy information about availability of data

All manuscripts must include a data availability statement. This statement should provide the following information, where applicable:
- Accession codes, unique identifiers, or web links for publicly available datasets
- A list of figures that have associated raw data
- A description of any restrictions on data availability

All relevant data are readily available from the authors.

Field-specific reporting

Please select the one below that is the best fit for your research. If you are not sure, read the appropriate sections before making your selection.

☒ Life sciences ☐ Behavioural & social sciences ☐ Ecological, evolutionary & environmental sciences

For a reference copy of the document with all sections, see nature.com/documents/nr-reporting-summary-flat.pdf

Life sciences study design

All studies must disclose on these points even when the disclosure is negative.

Sample size	For fluorescence intensity, we measured minimum of 100 cells from three independent experiments. For other experiments (e.g. WB, FACS, IP, in vitro methylation assay) were repeated three times.
Data exclusions	No data were excluded from the analysis.
Replication	All attempts at replication were successful.
Randomization	All samples were randomly allocated into experimental group.
Blinding	Four the blinding experiments, we masked the name of the samples and the quantifications were perfomed by a different person who did not perform the experiment.

Reporting for specific materials, systems and methods

We require information from authors about some types of materials, experimental systems and methods used in many studies. Here, indicate whether each material, system or method listed is relevant to your study. If you are not sure if a list item applies to your research, read the appropriate section before selecting a response.

Materials & experimental systems

n/a	Involved in the study	
☐	☒	Antibodies
☐	☒	Eukaryotic cell lines
☒	☐	Palaeontology and archaeology
☒	☐	Animals and other organisms
☒	☐	Human research participants
☒	☐	Clinical data
☒	☐	Dual use research of concern

Methods

n/a	Involved in the study	
☒	☐	ChIP-seq
☐	☒	Flow cytometry
☒	☐	MRI-based neuroimaging

Antibodies

Antibodies used

Format:
Antibody name (Species, Applications, Cat. NO., LOT. NO., Clone name/number, Company)
[Primary antibody]
anti-phospho Tyrosine (pY100)(mouse, WB, #9411, -,-, Cell Signaling Technology)
anti-pY416 Src kinase (rabbit, WB, #2101, -, -, Cell Signaling Technology)
anti-γH2AX (rabbit, WB/IF, #2577, -, -, Cell Signaling Technology)
anti-histone H3 (rabbit, WB, #9715, -, -, Cell Signaling Technology)
anti-histone H4 (rabbit, WB, #2592, -, -, Cell Signaling Technology)
anti-pCHK1 (rabbit, WB, #2344, -, -, Cell Signaling Technology)
anti-CHK1 (mouse, WB, #2360, -, 3G1D5, Cell Signaling Technology)
anti-pCHK2 (rabbit, WB, #2661, -, -, Cell Signaling Technology)
anti-CHK2 (rabbit, WB, #2662, -, -, Cell Signaling Technology)
anti-cleaved caspase-3 (rabbit, WB, #9661, -, -, Cell Signaling Technology)
anti-PRMT5 (rabbit, WB/IP, 07-405, -, -, Merck Millipore)
anti-cSrc kinase (mouse, WB/IP, 05-184, -, GD11, Merck Millipore)
anti-SDMA (Sym10) (rabbit, WB, 07-412, -, -, Merck Millipore)
anti-ADMA (Asym24) (rabbit, WB, 07-414, -, -, Merck Millipore)
anti-c-myc (mouse, WB/IP/IF, sc-40, -, 9E10, Santa Cruz Biotechnology, Inc.)
anti-β-actin (mouse, WB, sc-8432, -, C-2, Santa Cruz Biotechnology, Inc.)
anti-PARP (rabbit, WB, sc-8007, -, F-2, Santa Cruz Biotechnology, Inc.)
anti-GFP (rabbit, WB/IF, A-11122, -, -, Thermo Scientific Invitrogen)
anti-GFP (mouse, IP/IF, A-11120, -, -, Thermo Scientific Invitrogen)
anti-53BP1 (rabbit, WB/IP/IF, A300-272A, -, -, Bethyl Laboratories, Inc.)

anti-MEP50 (rabbit, WB/IP, A301-562A, -, -, Bethyl Laboratories, Inc.)
anti-H3R8me2s (rabbit, WB, ab130740, -, -, abcam)
anti-H4R3me2s (rabbit, WB, ab5823, -, -, abcam)
anti-H3R17me2a (rabbit, WB, ab8284, -, -, abcam)
anti-H4K20me2 (rabbit, WB/IF, ab9052, -, -, abcam)
[Secondary antibody]
anti-rabbit IgG-HRP conjugated (goat, WB, 115-035-003, -, -, Jackson ImmunoResearch Inc.)
anti-Mouse IgG-HRP conjugated (goat, WB, 111-035-003, -, -, Jackson ImmunoResearch Inc.)
DyLight® 594 goat anti-mouse IgG (goat, IF, A90-138D4, -, -, Bethyl Laboratories, Inc.)
FITC goat anti-rabbit IgG (goat, IF, A120-101F, -, -, Bethyl Laboratories, Inc.)

Validation

Format:
Antibody name (Species, Applications, Cat. NO., LOT. NO., Clone name/number, Company)
[Primary antibody]

anti-phospho Tyrosine (pY100)(mouse, WB, #9411, -,-, Cell Signaling Technology)
Species reactivity: human, mouse, rat
Application: WB/IP/IHC/IF/F
Citation: Li, S. et al. (2019), Early Histone Deacetylase Inhibition Mitigates Ischemia/Reperfusion Brain

anti-pY416 Src kinase (rabbit, WB, #2101, -, -, Cell Signaling Technology)
Species reactivity: Human, Mouse, Rat
Application: WB
Citation:Thomas, S.M. and Brugge, J.S. (1997) Annu Rev Cell Dev Biol 13, 513-609.

anti-γH2AX (rabbit, WB/IF, #2577, -, -, Cell Signaling Technology)
Species reactivity: Human, Mouse, Rat, Monkey
Application: WB/IF/F
Citation: Yuan, J. et al. (2010) FEBS Lett 584, 3717-24.

anti-histone H3 (rabbit, WB, #9715, -, -, Cell Signaling Technology)
Species reactivity: Human, Mouse, Rat, Monkey, Zebrafish, Bovine, Pig
Application: WB
Citation: Workman, J.L. and Kingston, R.E. (1998) Annu Rev Biochem 67, 545-79.

anti-histone H4 (rabbit, WB, #2592, -, -, Cell Signaling Technology)
Species reactivity: Human, Mouse, Rat, Monkey, D. melanogaster, Zebrafish, S. cerevisiae
Application: WB/IP
Citation: Hu, X., Xiang, D., et al. (2019) Oncogene, 1 October

anti-pCHK1 (rabbit, WB, #2344, -, -, Cell Signaling Technology)
Species reactivity: Human, Monkey, Mink
Application: WB
Citation: Liu, Q. et al. (2000) Genes Dev 14, 1448-59.

anti-CHK1 (mouse, WB, #2360, -, 3G1D5, Cell Signaling Technology)
Species reactivity: Human, Mouse, Rat, Monkey
Application: WB
Citation: Liu, Q. et al. (2000) Genes Dev 14, 1448-59.

anti-pCHK2 (rabbit, WB, #2661, -, -, Cell Signaling Technology)
Species reactivity: Human, Monkey
Application: WB/IP/IF/F
Citation: Allen, J.B. et al. (1994) Genes Dev. 8, 2401-2415.

anti-CHK2 (rabbit, WB, #2662, -, -, Cell Signaling Technology)
Species reactivity: Human, Mouse, Rat, Monkey
Application: WB/IP
Citation: Allen, J.B. et al. (1994) Genes Dev. 8, 2401-2415.

anti-cleaved caspase-3 (rabbit, WB, #9661, -, -, Cell Signaling Technology)
Species reactivity: Human, Mouse, Rat, Monkey
Application: WB/IP/IHC/IF/F
Citation: Fernandes-Alnemri, T. et al. (1994) J Biol Chem 269, 30761-4.

anti-PRMT5 (rabbit, WB/IP, 07-405, -, -, Merck Millipore)
Species reactivity: Human, Mouse
Application: WB/IP
Citation: Irie, N et al. Cell (2015) 160, 253-68.

anti-Src kinase (mouse, WB/IP, 05-184, -, GD11, Merck Millipore)
Species reactivity: Av, H, M, Mi, R
Application: WB/IP
Citation: Tabariès, S et al. (2015) Oncotarget, 6, 9476-87.

anti-SDMA (Sym10) (rabbit, WB, 07-412, -, -, Merck Millipore)
Species reactivity: Human, Mouse
Application: WB/IP/IF
Citation: Takai, H et al. (2014) Cell reports, 9, 48-60.

anti-ADMA (Asym24) (rabbit, WB, 07-414, -, -, Merck Millipore)
Species reactivity: Human, Mouse
Application: WB
Citation: Hussein et al. (2015) PloS one, 10, e0135218.

anti-c-myc (mouse, WB/IP/IF, sc-40, -, 9E10, Santa Cruz Biotechnology, Inc.)
Species reactivity: mouse, rat, human, monkey, feline and canine
Application: WB/IP/IHC/IF/F/ELISA
Citation: Petsalaki, E. et al. (2016) Nature communications, 7, 11451.

anti-β-actin (mouse, WB, sc-8432, -, C-2, Santa Cruz Biotechnology, Inc.)
Species reactivity: mouse, rat, human
Application: WB/IP/IHC/IF/F/ELISA
Citation: Jiang, Y. et al. (2019) Oncol. Rep.

anti-PARP (rabbit, WB, sc-8007, -, F-2, Santa Cruz Biotechnology, Inc.)
Species reactivity: human
Application: WB/IP/IHC/IF/ELISA
Citation: Lee, J. et al. 2019. Blood.

anti-GFP (rabbit, WB/IF, A-11122, -, -, Thermo Scientific Invitrogen)
Species reactivity: tag
Application: WB/IHC/IF/ICC
Citation: Front Cell Neurosci. (2017) Jan, 26, 11:6.

anti-GFP (mouse, IP/IF, A-11120, -, -, Thermo Scientific Invitrogen)
Species reactivity: tag
Application: IP/IF/ICC/ELISA
Citation: Front Cell Neurosci. (2018) Jul, 3;12:186.

anti-53BP1 (rabbit, WB/IP/IF, A300-272A, -, -, Bethyl Laboratories, Inc.)
Species reactivity: Human, Mouse
Application: WB/IP/IHC
Citation: Shimada, M., Tsukada, K., et al. (2019). 22 November

anti-MEP50 (rabbit, WB/IP, A301-562A, -, -, Bethyl Laboratories, Inc.)
Species reactivity: Human
Application: WB/IP/IHC
Citation: Menin epigenetically represses Hedgehog signaling in MEN1 tumor syndrome. Cancer Res (2013)

anti-H3R8me2s (rabbit, WB, ab130740, -, -, abcam)
Species reactivity: Cow, Human
Application: Peptide Array/IP/WB/IHC-P
Citation: Liu R et al. Nucleic Acids Res (2018) 46:6608-6626.

anti-H4R3me2s (rabbit, WB, ab5823, -, -, abcam)
Species reactivity: Mouse, Cow, Human, Saccharomyces cerevisiae, Xenopus laevis, Arabidopsis thaliana, Drosophila melanogaster, Tobacco
Application: IHC-P/Peptide Array/ICC/IF/WB
Citation: Demetriadou C et al. (2019) Cell Death Dis, 10:236

anti-H3R17me2a (rabbit, WB, ab8284, -, -, abcam)
Species reactivity: Mouse, Rat, Chicken, Cow, Human, Caenorhabditis elegans, Drosophila melanogaster, Toxoplasma gondii
Application: ChIP/Peptide Array/IHC-P/ICC/IF/Dot blot/IP/WB
Citation: Greenblatt SM et al. (2018) Cancer Cell, 33:1111-1127.e5

anti-H4K20me2 (rabbit, WB/IF, ab9052, -, -, abcam)
Species reactivity: Mouse, Cow, Human, Schizosaccharomyces pombe, Toxoplasma gondii
Application: IP/WB/ChIP/ICC/IF/IHC
Citation: Alyodawi K et al. (2019) J Cachexia Sarcopenia Muscle, 10:662-686

[Secondary antibody]

anti-rabbit IgG-HRP conjugated (goat, WB, 115-035-003, -,-, Jackson ImmunoResearch Inc.)
Species reactivity: rabbit
Application: WB
Citation: Orexin A-Mediated Modulation of Reproductive Activities in Testis of Normal and Cryptorchid Dogs: Possible Model for Studying Relationships Between Energy Metabolism and Reproductive Control. Loredana Assisi, Alessandra Pelagalli, Caterina Squillacioti, Giovanna Liguori, Chiara Annunziata, Nicola Mirabella, Frontiers in Endocrinology, 10 , p816

anti-Mouse IgG-HRP conjugated (goat, WB, 111-035-003, -,-, Jackson ImmunoResearch Inc.)

Species reactivity: rabbit
Application: WB
Citation: Orexin A-Mediated Modulation of Reproductive Activities in Testis of Normal and Cryptorchid Dogs: Possible Model for Studying Relationships Between Energy Metabolism and Reproductive Control. In Frontiers in Endocrinology on 12 December 2019 by Assisi, L., Pelagalli, A., et al..

DyLight® 594 goat anti-mouse IgG (goat, IHC/ICC/F/IF, A90-138D4, -, -, Bethyl Laboratories, Inc.)
Species reactivity: mouse
Application: IHC, ICC, F, IF
Citation: -

FITC goat anti-rabbit IgG (goat, IHC/ICC/F/IF, A120-101F, -, -, Bethyl Laboratories, Inc.)
Species reactivity: rabbit
Application: IHC, ICC, F, IF
Citation: Pro-apoptotic effect of the novel benzylidene derivative MHY695 in human colon cancer cells. Oncol Lett

Eukaryotic cell lines

Policy information about cell lines

Cell line source(s)	HEK293T, U2OS, MCF7, A549 , and NIH3T3 cells were purchased from ATCC.
Authentication	The Fingerprinting of all cell lines by 'AmplFLSTR identifiler PCR Amplification kit' was tested on July 12, 2019 in Korean Cell Line Bank.
Mycoplasma contamination	All cell lines tested negative for mycoplasma contamination.
Commonly misidentified lines (See ICLAC register)	No commonly misidentified cell lines were used.

Flow Cytometry

Plots

Confirm that:

☒ The axis labels state the marker and fluorochrome used (e.g. CD4-FITC).

☒ The axis scales are clearly visible. Include numbers along axes only for bottom left plot of group (a 'group' is an analysis of identical markers).

☒ All plots are contour plots with outliers or pseudocolor plots.

☒ A numerical value for number of cells or percentage (with statistics) is provided.

Methodology

Sample preparation	Cells were harvested by trypsinization and washed with PBS. Living cells were analyzed.
Instrument	BD FACS Calibur 220 AVR
Software	BD CellQuest™ Pro Software
Cell population abundance	Living cells in PBS were gated by FSC/SSC using CellQuest™ Pro Software
Gating strategy	GFP-positive cells were gated by FL1 intensity

☐ Tick this box to confirm that a figure exemplifying the gating strategy is provided in the Supplementary Information.
