# Supplementary material for: PRMT5 promotes DNA repair through methylation of 53BP1 and is regulated by Src-mediated phosphorylation
Source: Commun Biol. 2020 Aug 5;3:428. doi: 10.1038/s42003-020-01157-z (PMC7406651; doi:10.1038/s42003-020-01157-z)
Supplement: Supplementary file 5 — Peer review [file 42003_2020_1157_MOESM5_ESM.pdf]

Reviewers' comments:

Reviewer #1 (Remarks to the Author):

Hwang et al show that PRMT5 plays a critical role in HR and NHEJ DNA repair in response to DNA damage. It does so by methylating and thereby stabilizing 53BP1, which plays a critical role in DNA damage repair. They also show that PRMT5 activity is negatively regulated by src-mediated phosphorylation of PRMT5. Since src activity is induced by DNA damage, the src mediated inhibition of PRMT5 activity seems contradictory to the other parts of the model. However, the authors show that PRMT5 methylation only occurs at very high etoposide concentrations where apoptosis is occurring. Thus, they propose that src-mediated PRMT5 phosphorylation serves as a system to sense the degree of DNA damage and to decide whether cells should pursue DNA repair and survive or block DNA repair and undergo apoptosis.

This is a very interesting paper with novel findings that potentially move the field forward. The data presented is for the most part consistent with the authors’ conclusions and models. However, the manuscript lacks sufficient explanations for many of the technical aspects of the paper, making it very difficult to read and in some cases difficult to understand how experiments were performed. In addition, reproducibility is not discussed or demonstrated in many of the figures. These problems in scientific rigor make it difficult to validate the authors’ conclusions. Detailed comments are below:

1) The data and methods for some experiments are not sufficiently described to allow readers to understand the significance of the experiment or how the experiment was performed.
a) In Fig 1 why is vsrc used in some experiments instead of c-src? The latter would be more relevant.
b) In Fig S1b the homology is very low, so it is not clear how the alignment was done, and therefore the conclusions drawn on the basis of homology (or lack of) are not convincing. Also, the different coloring of amino acids is not explained.
c) In S1e what is GRAA and why is it being used in this experiment?
d) In S1d what is the significance of the results with the pY100 antibody?
e) In S1h what is the significance of the results with the GRAA mutant?
f) The DR-GFP and Ej5-GFP experiments are not sufficiently described in the results or methods, and no references are given for the assays or the cell lines. The I-SceI plasmid and protein are not described and are mis-spelled in Fig 5a&b and S4a&b.

2) While statistical significance and reproducibility are generally addressed in the graphs, figures containing western blots and immunofluorescence where data from only a single experiment are presented lack any demonstration or discussion of reproducibility. In some cases the data shown are not consistent with the description of the data in Results.
a) Reproducibility of all results that show data from a single experiment needs to be discussed or demonstrated.
b) In 3f it appears that low (10 uM) etoposide actually induces histone methylation, while higher concentrations repress it.
c) In S2d the conclusion that pCHK2 is reduced by PRMT5 depletion is not convincingly shown in the data. The differences are very small, and reproducibility is not discussed or demonstrated.
d) in Fig 4b & c it would be more accurate to say that SDMA and ADMA were “reduced”, since the statement that they were “abolished” is not supported by the data shown.
e) In Fig 4e the differences in 53BP1 levels are small and not sufficiently documented by statistical analysis of multiple experiments.
f) In S4c and 5e the differences described are small and not convincing since there is no statistical analysis or demonstration of reproducibility.

3) The model in Fig S5e is good, important for the manuscript, and should be moved to Fig 5.

Reviewer #2 (Remarks to the Author):

The current manuscript investigated the role of PRMT5, a type-2 arginine methyltransferase, in DNA damage response (DDR) and its regulation by the Src kinase. The results showed that PRMT5 is phosphorylated by the Src kinase at Y324 residue in vitro and in vivo, which led to attenuation of its methyltransferase activity as evidenced by the reduction of histone arginine methylation. PRMT5 Y324 phosphorylation occurred in vivo upon etoposide treatment. This is consistent with previous findings that PRMT5 is involved in DDR. In addition, PRMT5 inhibition by EPZ015666 or Y324F mutation led to 53BP1 protein instability and defects in DNA damage repair. The authors concluded that Src mediated PRMT5 phosphorylation suppresses its ability to stabilize 53BP1.

Overall, this study revealed a new regulation of PRMT5 by the Src kinase and additional mechanism of PRMT5 in DDR. The results are quite interesting although further clarifications are necessary to convince the audience.

Specific Comments:
1. In vivo analyses of etoposide induced pY324 were generated by overexpression of myc-PRMT5. It should be examined whether endogenous PRMT5 Y324 phosphorylated can be detected upon DDR.
2. It should be addressed whether PRMT5 Y324 phosphorylation or its methyltransferase activity affect histone arginine methylation (e.g. H3R8me2, H4R3me2) as well as lysine methylation (H4K20me, required for 53BP1 recruitment and foci formation) locally at DNA damage sites.
3. SDMA of 53BP1-tudor was attenuated in 5RT mutant but remained clearly visible. Similarly, siPRMT5 did not completely abolish SDMA of 53BP1-tudor, which might be due to partial depletion of PRMT5. The choice of language should be toned down to reflect the results accurately.
4. Results showed EPZ015666 or PRMT5 mutants decreasing 53BP1 protein stability. It should be clarified if EPZ015666 or PRMT5 mutants affect cell cycle or cell viability which indirectly influence 53BP1 protein stability.
5. Fig 4g is not very convincing. It can be replaced by Fig S3g and Fig S3h.
6. Fig 4h showed that PRMT5 Y324F mutant attenuated 53BP1 foci, probably due to 53BP1 protein instability. This interesting observation however contradicted with two recent publications that PRMT5 depletion or knockout enhanced 53BP1 foci upon IR (Clarke et al., 2017, Molecular Cell 65, 900–916; Hamard et al., 2018, Cell Reports 24, 2643–2657).
7. The image quality of Fig 5d was poor and cannot be assessed properly. Additionally, gammaH2AX should be analyzed quantitatively by foci kinetics.
8. Fig 5f, clonogenic survival should be assessed with a titration of DNA damaging agent to differentiate sensitivities of wild type cells verse mutant cells based on proper calculation of surviving colonies.
9. Regulation of 53BP1 protein stability in DDR has been previously investigated by multiple groups. Those previous results and possible intersection with PRMT5 should be discussed.

Minor points:
1. Induction of PRMT5 pY324 upon various DNA damaging agents (camptothecin, doxorubicin, and UV) should be included in supplementary information.
2. Fig4a, the amino acid coverage of 53BP1-tudor domain, GAR deletion, and positions of 5RK should be indicated in the figure legend.

*We thank the reviewers for their criticisms and suggestions. We addressed all the specific comments below through additional experiments and made changes in the text and provided new figures. We greatly appreciate the reviewers' comments, as the changes that have been made strengthened our conclusions. For clarity, we include **reviewers' comments in black** and **our responses in blue**.*

Reviewers' comments:

Reviewer #1 (Remarks to the Author):

Hwang et al show that PRMT5 plays a critical role in HR and NHEJ DNA repair in response to DNA damage. It does so by methylating and thereby stabilizing 53BP1, which plays a critical role in DNA damage repair. They also show that PRMT5 activity is negatively regulated by src-mediated phosphorylation of PRMT5. Since src activity is induced by DNA damage, the src mediated inhibition of PRMT5 activity seems contradictory to the other parts of the model. However, the authors show that PRMT5 methylation only occurs at very high etoposide concentrations where apoptosis is occurring. Thus, they propose that src-mediated PRMT5 phosphorylation serves as a system to sense the degree of DNA damage and to decide whether cells should pursue DNA repair and survive or block DNA repair and undergo apoptosis.

This is a very interesting paper with novel findings that potentially move the field forward. The data presented is for the most part consistent with the authors' conclusions and models. However, the manuscript lacks sufficient explanations for many of the technical aspects of the paper, making it very difficult to read and in some cases difficult to understand how experiments were performed. In addition, reproducibility is not discussed or demonstrated in many of the figures. These problems in scientific rigor make it difficult to validate the authors' conclusions. Detailed comments are below:

1) The data and methods for some experiments are not sufficiently described to allow readers to understand the significance of the experiment or how the experiment was performed.

→ We appreciate your helpful comments. According to your comments, we improved our MS by adding new experiments and more detailed explanation of our data and methods.

a) In Fig 1 why is vsrc used in some experiments instead of c-src? The latter would be more relevant.

→ As you know, vSrc is constitutively active form of cSrc kinase. Because vSrc strongly phosphorylated PRMT5 compared to cSrc (see the Fig), we used, in this study, vSrc to investigate the role or Y phosphorylation of PRMT5 more clearly.

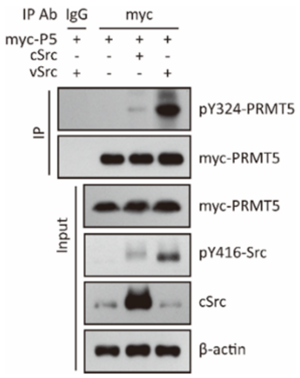

b) In Fig S1b the homology is very low, so it is not clear how the alignment was done, and therefore the conclusions drawn on the basis of homology (or lack of) are not convincing. Also, the different coloring of amino acids is not explained.

→ The alignment analysis in Fig S1b was performed by COBALT (Constraint-based Multiple Alignment Tool, https://www.ncbi.nlm.nih.gov/tools/cobalt) database. PRMT family members harbor a set of four conserved sequence motifs (I, post-I, II, and III) and a THW loop. FigS1b shows the front part of Motif I. The red color indicates highly conserved columns and blue indicates less conserved ones. This explanation was added to the Fig S1b legend.

c) In S1e what is GRAA and why is it being used in this experiment?

→ GRAA is an enzyme dead form of PRMT5 where the G367-R368 residues are substituted with alanine, respectively. Fig S1e data indicates that tyrosine phosphorylation by Src kinase occurs even in GRAA mutants with intact Y324 residues. We added this explanation in Page 6.

d) In S1d what is the significance of the results with the pY100 antibody?

→ We developed an antibody that selectively recognizes the phosphorylated Y324 PRMT5 (pY324-PRMT5). In Fig S1d, we compared the performance of pY324-PRMT5 antibody with pY100. Compared to the pY100 antibody, our pY324-PRMT5 antibody recognized more effectively the PRMT5 phosphorylation by Src kinase, which was described in Page 6.

e) In S1h what is the significance of the results with the GRAA mutant?
→ As mentioned above comment c), GRAA is an enzyme dead form of PRMT5, so we used GRAA as a negative control in experiments to measure PRMT5 enzyme activity. Fig S1h shows that all Y324 mutants used have weak enzymatic activity, which are comparable to the GRAA mutant.

f) The DR-GFP and Ej5-GFP experiments are not sufficiently described in the results or methods, and no references are given for the assays or the cell lines. The I-SceI plasmid and protein are not described and are mis-spelled in Fig 5a&b and S4a&b.

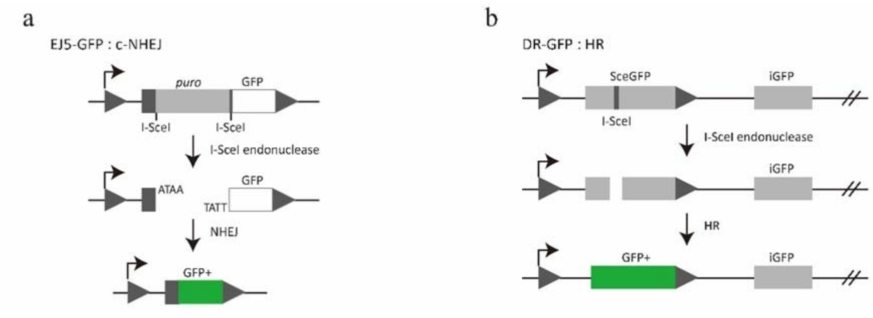

→ More detailed experimental methods and citations for DR-GFP and EJ5-GFP experiments were added (Fig. S6a and b). In addition, we corrected typo you pointed out in Fig 5 and S4 (I-SecI → I-SceI).

2) While statistical significance and reproducibility are generally addressed in the graphs, figures containing western blots and immunofluorescence where data from only a single experiment are presented lack any demonstration or discussion of reproducibility. In some cases the data shown are not consistent with the description of the data in Results.

→ Thank you for your critical comments. According to your comments, we improved our MS by adding new experiments and clearer explanation/processing of our data.

a) Reproducibility of all results that show data from a single experiment needs to be discussed or demonstrated.

→ To address the reproducibility issue you raised, the results were quantified and graphed through 3-independent experiments (Fig4d, 4e, 4f, 5e, and S6e). All of our immunofluorescence experiments have already been quantified and statistically processed through repeated experiments.

b) In 3f it appears that low (10 uM) etoposide actually induces histone methylation, while higher concentrations repress it.

→ To address the issue you pointed out, we repeated this experiment and obtained the same result; a slight increase in H3R8me2s and H4R3me2s at low concentrations of etoposide (10 uM). In this situation, the PRMT5 and its co-activator MEP50 protein expression were no significant difference. The decrease in H3R8me2s/H4R3me2s at high concentration of etoposide can be explained by the inhibition of PRMT5 activity due to the Y324 phosphorylation, but the increase at low concentration is still unexplainable. However, it is possible that PRMT5 activity might be increased to promote DNA repair process in weak damage condition, and the responsible mechanism has not been reveled at this time.

g

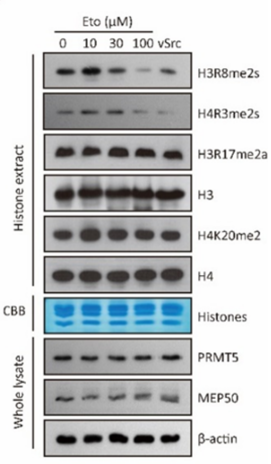

c) In S2d the conclusion that pCHK2 is reduced by PRMT5 depletion is not convincingly shown in the data. The differences are very small, and reproducibility is not discussed or demonstrated.

→ To address this point, we repeated this experiment several times, and concluded that the level of pChk2 is reduced by PRMT5 depletion. We switched it with a more explicit blot (Supplementary Fig. 3a).

a

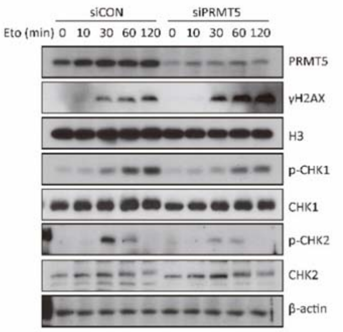

d) in Fig 4b & c it would be more accurate to say that SDMA and ADMA were “reduced”, since the statement that they were “abolished” is not supported by the data shown.
→ According to your suggestion, we corrected the expression from 'abolished' to 'reduced' in Page 10.

e) In Fig 4e the differences in 53BP1 levels are small and not sufficiently documented by statistical analysis of multiple experiments.

→ The 53BP1 level in Fig 4e (→Fig 4f) was quantified and graphed through 3 independent experiments.

f

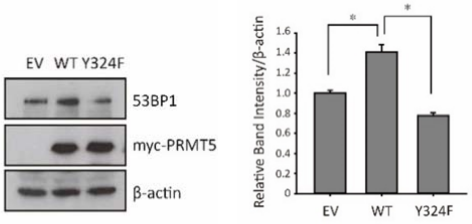

f) In S4c and 5e the differences described are small and not convincing since there is no statistical analysis or demonstration of reproducibility.

e

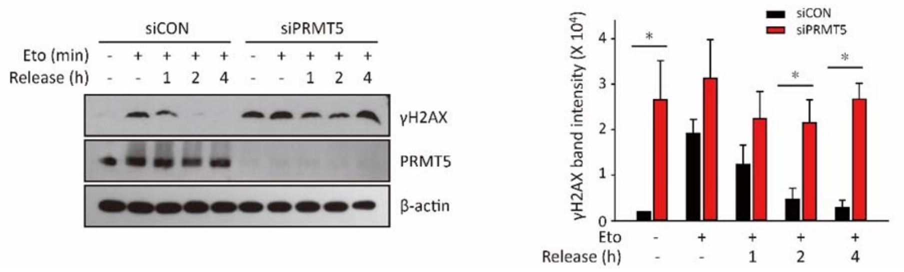

f

e

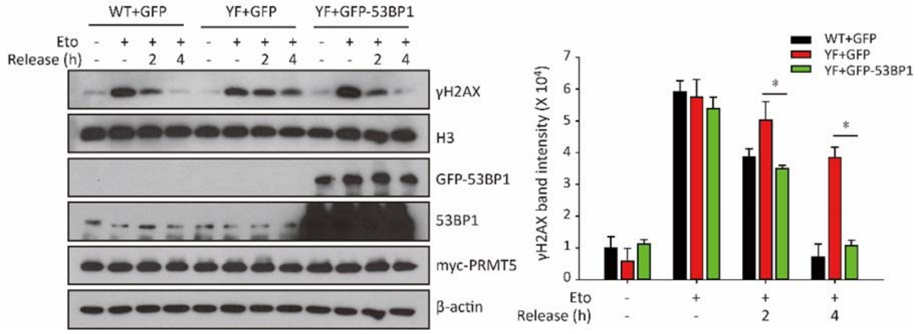

→ The γH2AX levels in Fig. S4c (→S6c) and Fig. 5e were quantified and graphed through 3 independent experiments.

3) The model in Fig S5e is good, important for the manuscript, and should be moved to Fig 5.

e

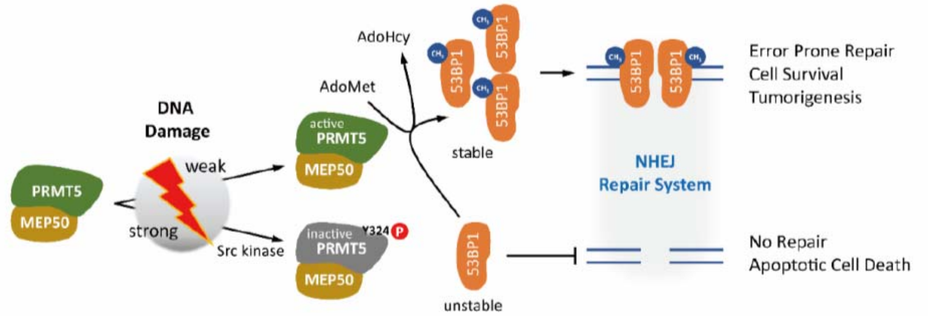

→ According to your suggestion, we moved the model from the supplementary S5e to Fig 6c.

Reviewer #2 (Remarks to the Author):

The current manuscript investigated the role of PRMT5, a type-2 arginine methyltransferase, in

DNA damage response (DDR) and its regulation by the Src kinase. The results showed that PRMT5 is phosphorylated by the Src kinase at Y324 residue in vitro and in vivo, which led to attenuation of its methyltransferase activity as evidenced by the reduction of histone arginine methylation. PRMT5 Y324 phosphorylation occurred in vivo upon etoposide treatment. This is consistent with previous findings that PRMT5 is involved in DDR. In addition, PRMT5 inhibition by EPZ015666 or Y324F mutation led to 53BP1 protein instability and defects in DNA damage repair. The authors concluded that Src mediated PRMT5 phosphorylation suppresses its ability to stabilize 53BP1.

Overall, this study revealed a new regulation of PRMT5 by the Src kinase and additional mechanism of PRMT5 in DDR. The results are quite interesting although further clarifications are necessary to convince the audience.

→ Thank you very much for your valuable comments and giving us the opportunity to revise our MS. According to your comments, we improved our MS by adding new experiments and more detailed explanation of our data.

Specific Comments:

1. In vivo analyses of etoposide induced pY324 were generated by overexpression of myc-PRMT5. It should be examined whether endogenous PRMT5 Y324 phosphorylated can be detected upon DDR.

→ To address this comment, we checked the Y324 phosphorylation of endogenous PRMT5 upon DDR. To do this experiment, we analyzed pY324 level of PRMT5 co-IP with MEP50 antibody, a co-activator of PRMT5, because of the very low immunoprecipitation efficiency of PRMT5. As shown in Fig 3d (new data), the Y324 phosphorylation was detected in endogenous PRMT5 co-IPed with MEP50.

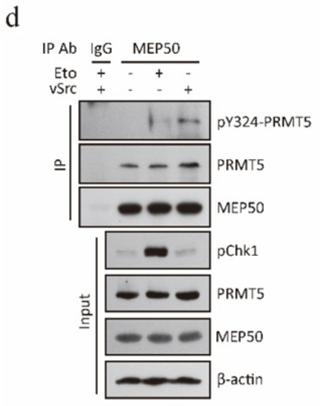

2. It should be addressed whether PRMT5 Y324 phosphorylation or its methyltransferase activity affect histone arginine methylation (e.g. H3R8me2, H4R3me2) as well as lysine methylation (H4K20me, required for 53BP1 recruitment and foci formation) locally at DNA damage sites.

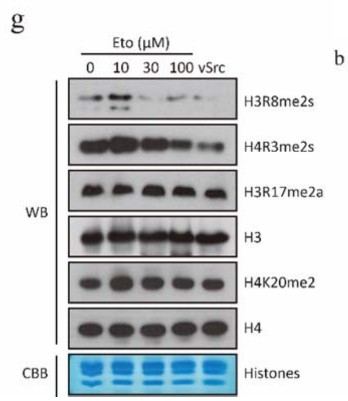

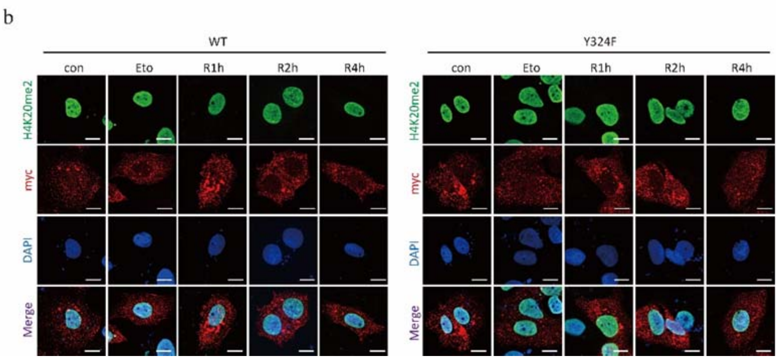

→ According to your comments, we checked the H4K20me2 level in response to etoposide treatment. The H4K20me2 level was not changed upon etoposide treatment, which was evidenced

by both western blot (Fig 3f, newly added) and the fluorescence imaging of nuclear H4K20me2 (Fig S7b, newly added). It was described in Page 9, 12.

3. SDMA of 53BP1-tudor was attenuated in 5RT mutant but remained clearly visible. Similarly, siPRMT5 did not completely abolish SDMA of 53BP1-tudor, which might be due to partial depletion of PRMT5. The choice of language should be toned down to reflect the results accurately.

→ According to your comment, we corrected the expression from 'abolished' to 'reduced' in Page 10.

4. Results showed EPZ015666 or PRMT5 mutants decreasing 53BP1 protein stability. It should be clarified if EPZ015666 or PRMT5 mutants affect cell cycle or cell viability which indirectly

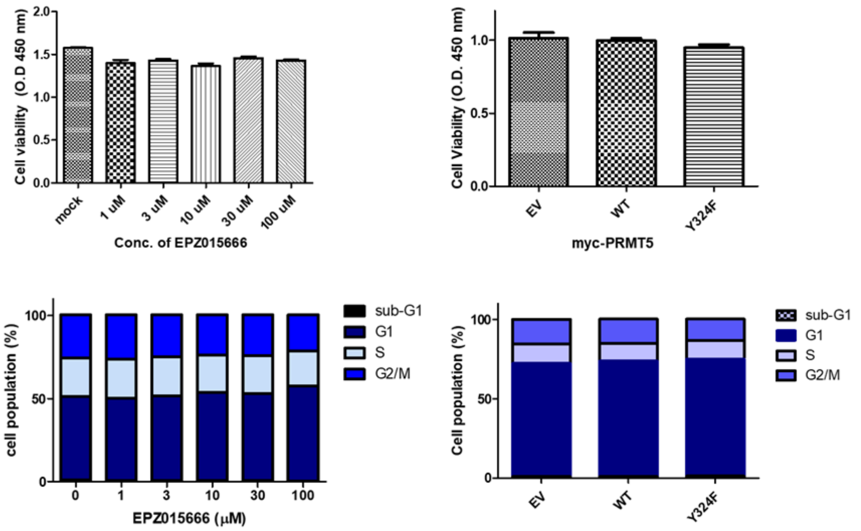

influence 53BP1 protein stability.

→ To address the issue you raised, we checked whether cell cycle or cellular viability is affected by EPZ015666 treatment or by Y324F transfection. As a result, we confirmed that both EPZ treatment and Y324 transfection did not affect cell cycle and cellular viability. This result was added to Fig. S5a-d, and described in Page 11.

5. Fig 4g is not very convincing. It can be replaced by Fig S3g and Fig S3h.
→ According to your comment, we replaced Fig 4g with Fig S3g.

6. Fig 4h showed that PRMT5 Y324F mutant attenuated 53BP1 foci, probably due to 53BP1 protein instability. This interesting observation however contradicted with two recent publications that PRMT5 depletion or knockout enhanced 53BP1 foci upon IR (Clarke et al., 2017, Molecular Cell 65, 900–916; Hamard et al., 2018, Cell Reports 24, 2643–2657).

→ As you mentioned above, recent reports show that PRMT5 depletion or knockout enhanced 53BP1 foci upon IR. In our experiment, PRMT5 depletion or Y324F expression did not affect the formation of 53BP1 foci very much, if we count the foci with even weak intensity, which is similar to the above results. However, the overall intensity and the number of high intensity 53BP1 foci were significantly decreased (Fig 4i), which shows a discrepancy from the above results. Unfortunately, we

cannot provide a clear explanation for this discrepancy at this time. However, based on our reproducible results, we believe that it is quite a reasonable explanation that the intensity of 53BP1 foci is governed by its stability regulation by PRMT5.

7. The image quality of Fig 5d was poor and cannot be assessed properly. Additionally, gammaH2AX should be analyzed quantitatively by foci kinetics.

d

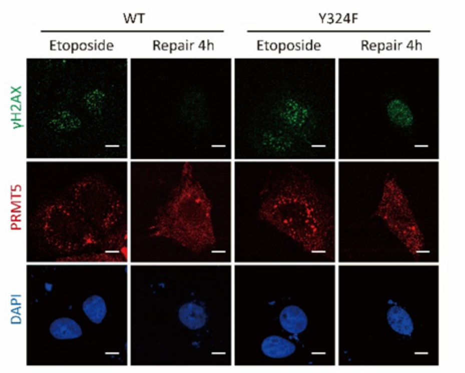

a

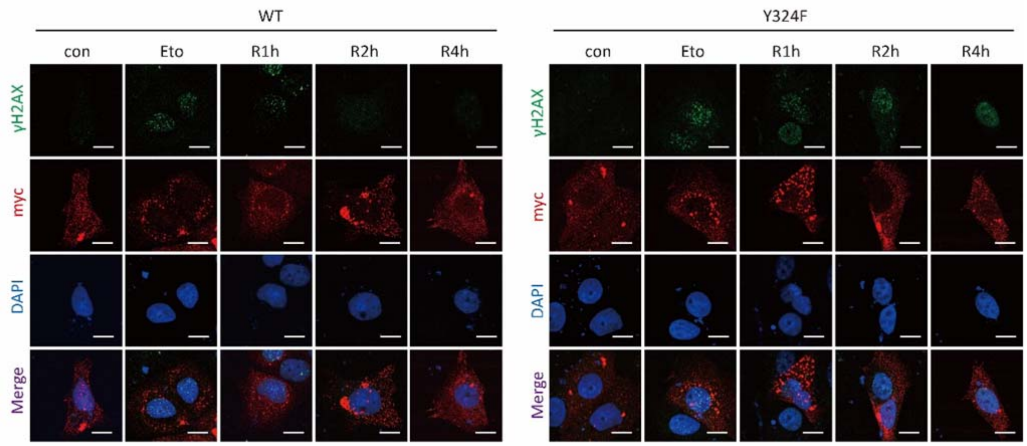

→ As recommended, we replaced Fig5d with a better resolution image (full version in Fig S7a) and analyzed the gammaH2AX level quantitatively by foci kinetics (already added this data to the figure Fig 5d right panel), which were described in Page 12.

8. Fig 5f, clonogenic survival should be assessed with a titration of DNA damaging agent to differentiate sensitivities of wild type cells verse mutant cells based on proper calculation of surviving colonies.

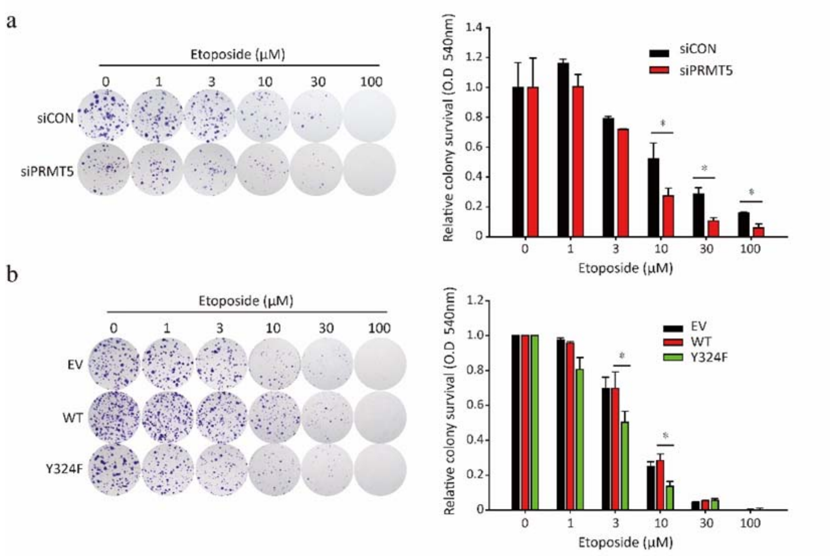

→ To address your comments, we performed colony forming assay with a titration of Etoposide and added the results to Fig 6a and b.

9. Regulation of 53BP1 protein stability in DDR has been previously investigated by multiple groups. Those previous results and possible intersection with PRMT5 should be discussed.
→ As pointed out, we described in discussion section as [It has been reported that ubiquitin-conjugating enzyme H7 (UbcH7, an ubiquitin E2 enzyme)[52], ring finger protein RNF8/RNF168 (both E3 ligases)[35] and Nudix hydrolase NUDT16[53] are involved in proteosomal degradation of 53BP1, respectively. Nevertheless, the mechanisms that regulate protein stability of 53BP1 are still not well understood. Further studies are needed to clarify the correlation between PRMT5-mediated arginine methylation and RNF8/RNF168-mediated ubiquitination or NUDT16-mediated ADP-ribosylation.].

Minor points:

1. Induction of PRMT5 pY324 upon various DNA damaging agents (camptothecin, doxorubicin, and UV) should be included in supplementary information.

→ According to your suggestions, the results of various DNA damaging agents were included in Fig S2c-f.

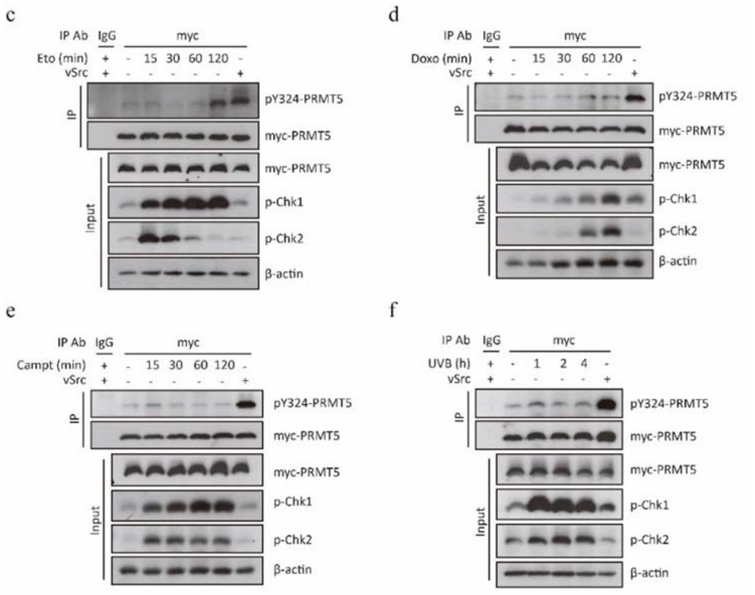

2. Fig4a, the amino acid coverage of 53BP1-tudor domain, GAR deletion, and positions of 5RK should be indicated in the figure legend.

→ According to your comments, we indicated the amino acid coverage of 53BP1-tudor domain, GAR deletion, and positions of 5RK in Fig S4a and described it in the legend.

a

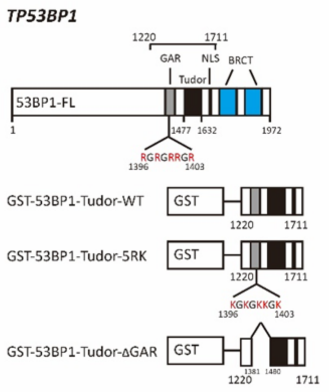

REVIEWERS' COMMENTS:

Reviewer #1 (Remarks to the Author):

The authors have generally addressed my previous criticisms. However, I still have a few comments for the revised manuscript.

I think the abstract does not sufficiently describe the role of Src. The model in Figure 6c and the Discussion suggest that Src phosphorylates and inhibits PRMT5 only in response to very strong DNA damage, to trigger apoptosis. As currently written, the Abstract makes it sound like Src contributes to cell survival by phosphorylating PRMT5, which is contradictory to the authors' findings and the rest of the model and abstract.

For many western blots, I still did not find any statements in the methods or figure legends where the authors have addressed the reproducibility of the results.

In Supp Fig 2 d-f the phosphorylation of PRMT5 is not convincing, and reproducibility is not discussed.

In Results, the sentence "However, we found that overexpression of wild type or Y324F PRMT5 did not unaltered the levels of phosphorylated CHK1or CHK2 (SupplementaryFig. 3b)." obviously needs to be rewritten.

Reviewer #2 (Remarks to the Author):

The revision has addressed the deficiencies.

We thank the reviewer for their criticisms and suggestions. We addressed all the specific comments below through additional descriptions and made changes in the text. We greatly appreciate the reviewer' comments, as the changes that have been made strengthened our conclusions. For clarity, we include ***reviewer' comments in black*** *and* ***our responses in blue****.*

Reviewer' comments:

Reviewer #1 (Remarks to the Author):

The authors have generally addressed my previous criticisms. However, I still have a few comments for the revised manuscript.

I think the abstract does not sufficiently describe the role of Src. The model in Figure 6c and the Discussion suggest that Src phosphorylates and inhibits PRMT5 only in response to very strong DNA damage, to trigger apoptosis. As currently written, the Abstract makes it sound like Src contributes to cell survival by phosphorylating PRMT5, which is contradictory to the authors' findings and the rest of the model and abstract.

→ Thank you for your considerable comment. As you pointed out, it sounds a little awkward. In order to make our conclusions clearer, we have made minor changes in Abstract as follows:

"Additionally, we show that PRMT5 activity is associated with non-homologous end joining (NHEJ) repair by methylation and stabilization of p53-binding protein 1 (53BP1), which contributes to cellular survival after DNA damage. Src-mediated phosphorylation of PRMT5 and subsequent inhibition of its activity in DNA damage process block NHEJ repair, leading to apoptotic cell death. Altogether, our findings suggest that PRMT5 regulates DNA repair process through Src-mediated Y324 phosphorylation in response to DNA damage."

For many western blots, I still did not find any statements in the methods or figure legends where the authors have addressed the reproducibility of the results.

→ All of our western blots are representative from at least three independent experiments. According to your comments, we mentioned the reproducibility of the results in corresponding figure legends and added "Statistics and Reproducibility" section in Materials and Methods.

In Supp Fig 2 d-f the phosphorylation of PRMT5 is not convincing, and reproducibility is not discussed.

→ To investigate whether PRMT5 is phosphorylated under DNA damage stress, we used etoposide, doxorubicin, camptothecin, and UVB as shown in Supplementary Fig. 2. Among them, the phosphorylation by etoposide was clearly observed. As you pointed out, although phosphorylation by doxorubicin, camptothecin and UVB was relatively weak, we obviously confirmed this phosphorylation of PRMT5 through several repeated experiments.

In Results, the sentence "However, we found that overexpression of wild type or Y324F PRMT5 did not unaltered the levels of phosphorylated CHK1or CHK2 (SupplementaryFig. 3b)." obviously needs to be rewritten.

→ Thank you for pointing out our obvious typo. We corrected the sentence, "However, we found that overexpression of wild type or Y324F PRMT5 did not alter the levels of phosphorylated CHK1or CHK2 (Supplementary Fig. 3b).".
